# Astroglial TLR9 antagonism promotes chemotaxis and alternative activation of macrophages via modulation of astrocyte-derived signals: implications for spinal cord injury

**DOI:** 10.1186/s12974-020-01748-x

**Published:** 2020-02-25

**Authors:** Lun Li, Li Ni, Robert F. Heary, Stella Elkabes

**Affiliations:** Reynolds Family Spine Laboratory, Department of Neurosurgery, New Jersey Medical School, Rutgers, The State University of New Jersey, 205 South Orange Avenue, F-1204, Newark, NJ 07103 USA

**Keywords:** Innate immune receptors, Toll-like receptor, Spinal cord injury, Astrocyte, Macrophage, Microglia, Chemokine, Cytokine, Chemotaxis

## Abstract

**Background:**

The recruitment of immune system cells into the central nervous system (CNS) has a profound effect on the outcomes of injury and disease. Glia-derived chemoattractants, including chemokines, play a pivotal role in this process. In addition, cytokines and chemokines influence the phenotype of infiltrating immune cells. Depending on the stimuli present in the local milieu, infiltrating macrophages acquire the classically activated M1 or alternatively activated M2 phenotypes. The polarization of macrophages into detrimental M1 versus beneficial M2 phenotypes significantly influences CNS pathophysiology. Earlier studies indicated that a toll-like receptor 9 (TLR9) antagonist modulates astrocyte-derived cytokine and chemokine release. However, it is not known whether these molecular changes affect astrocyte-induced chemotaxis and polarization of macrophages. The present studies were undertaken to address these issues.

**Methods:**

The chemotaxis and polarization of mouse peritoneal macrophages by spinal cord astrocytes were evaluated in a Transwell co-culture system. Arrays and ELISA were utilized to quantify chemokines in the conditioned medium (CM) of pure astrocyte cultures. Immunostaining for M1- and M2-specific markers characterized the macrophage phenotype. The percentage of M2 macrophages at the glial scar was determined by stereological approaches in mice sustaining a mid-thoracic spinal cord contusion injury (SCI) and intrathecally treated with oligodeoxynucleotide 2088 (ODN 2088), the TLR9 antagonist. Statistical analyses used two-tailed independent-sample *t*-test and one-way analysis of variance (ANOVA) followed by Tukey’s *post hoc* test. A *p* value < 0.05 was considered to be statistically significant.

**Results:**

ODN 2088-treated astrocytes significantly increased the chemotaxis of peritoneal macrophages via release of chemokine (C-C motif) ligand 1 (CCL1). Vehicle-treated astrocytes polarized macrophages into the M2 phenotype and ODN 2088-treated astrocytes promoted further M2 polarization. Reduced CCL2 and CCL9 release by astrocytes in response to ODN 2088 facilitated the acquisition of the M2 phenotype, suggesting that CCL2 and CCL9 are negative regulators of M2 polarization. The percentage of M2 macrophages at the glial scar was higher in mice sustaining a SCI and receiving ODN 2088 treatment as compared to vehicle-treated injured controls.

**Conclusions:**

TLR9 antagonism could create a favorable environment during SCI by supporting M2 macrophage polarization and chemotaxis via modulation of astrocyte-to-macrophage signals.

## Background

Central nervous system (CNS) injury and diseases are often associated with the infiltration of immune cells into the CNS. These cells play critical roles in neuroinflammation, neurodegeneration, and paradoxically, neuroprotection [1]. The recruitment of immune cells into the affected sites is mediated by chemoattractants secreted by various cell types including those intrinsic to the CNS [2]. Astrocytes are one of the cell types that secrete potent chemoattractants [3, 4] such as chemokines. Peripheral macrophages are among immune system cells that are recruited to the CNS during disease and injury [5]. Macrophages show phenotypic plasticity depending on the environmental signals that are present at the affected sites [6]. Under the influence of effectors such as interferon-gamma (IFN-γ), macrophages acquire the classically activated M1 phenotype [7], which has been associated with detrimental effects [8], whereas effectors such as interleukin 4 (IL-4), IL-13, and transforming growth factor beta (TGF-β) promote the alternatively activated M2 phenotype, which has been implicated in protective and beneficial effects [9]. The M1/M2 ratio can be an important determinant of outcomes in CNS pathology [10].

Astrocytes play many fundamental roles in health and disease, including maintenance of homeostasis [11], modulation of neuronal and synaptic function [12], repair of the blood-brain barrier [13], and protection of neurons from insults [14]. Astrocytes express toll-like receptors (TLRs) [15–17], which are pattern recognition receptors (PRRs) that are best known for their role in the innate immune system in response to infections. TLRs are the first sensors of danger and recognize conserved molecular motifs in pathogens collectively called pathogen-associated molecular patterns (PAMPs). Binding of PAMPS to TLRs initiates an immune reaction to protect the host from pathogens. In addition, TLRs are important contributors to sterile inflammation that occurs in injury and diseases. In this case, endogenous ligands released by stressed, damaged, or dying cells, called danger-associated molecular patterns (DAMPs), initiate TLR signaling [18]. In addition to PAMPs and DAMPs, the availability of synthetic stimulatory or inhibitory TLR ligands has facilitated research in experimental conditions [19].

TLRs play key roles in traumatic brain and spinal cord injury (SCI) [20, 21], multiple sclerosis [22], epilepsy [23], and neuropathic pain [24]. Several TLRs have been identified in the mouse and humans [25]. Genetic deletion, stimulation, or inhibition of TLRs has led to amelioration or worsening of deficits in animal models of CNS pathology [26, 27], depending on the TLR, the ligand utilized, and the pathological condition investigated. This led to the conclusion that TLRs play distinct roles in the CNS, albeit further studies are necessary to elucidate the precise and unique role played by each TLR.

Studies in our laboratory have focused on TLR9. We have shown that a TLR9 antagonist, oligodeoxynucleotide 2088 (ODN 2088), alleviates functional deficits and improves histopathology in mice sustaining an SCI [28, 29]. We also reported that TLR9 immunoreactivity and mRNA are found in astrocytes, in vitro [15] and in vivo [30]. Direct inhibition of TLR9 in pure spinal cord (SC) astrocyte cultures alters the release of cytokines and chemokines [15] raising the possibility that astroglial TLR9 antagonism could modulate astrocyte-to-macrophage signals and this, in turn, could alter astrocyte-induced chemotaxis and polarization of macrophages.

The present investigations were undertaken to determine whether ODN 2088 modulates astrocyte-induced chemotaxis of macrophages, in an in vitro Transwell system that enables co-culture of pure SC astrocytes with peritoneal macrophages. We also determined whether astrocyte-derived signals modulate the phenotypic polarization of macrophages, in vitro and the percentage of M2 macrophages at the injury epicenter following SCI.

## Methods

### Animals

Adult and postnatal day 3, C57BL/6 female mice were obtained from Charles River Laboratories (Wilmington, MA, USA). TLR9^−/−^ mice on C57BL/6 background were bred at New Jersey Medical School, Rutgers, The State University of New Jersey. All mice were housed in a pathogen-free barrier facility on a 12:12 h light-dark cycle with water and food provided ad libitum. Sentinels were housed in the same room and periodically checked for infection. All animal protocols and experiments were approved by the Institutional Animal Care and Use Committee (IACUC) at Rutgers University and were performed in accordance with relevant guidelines and regulations.

### Preparation of spinal cord astrocyte cultures

Three-day-old mouse pups were euthanized by exposure to CO_2_ followed by decapitation. The SCs were dissected and the meninges were carefully removed. The tissue was dissociated by trituration and the cells were plated in poly-d-lysine (Sigma-Aldrich, St. Louis, MO, USA) coated 75 cm^2^ culture flasks in minimum essential medium (MEM; Thermo Fisher Scientific, Waltham, MA, USA) supplemented with 15% heat-inactivated fetal bovine serum (FBS; Thermo Fisher Scientific) and 10 units/ml penicillin/streptomycin (NM-15). Cell suspensions obtained from three SCs were plated in each flask. The culture media were removed and fresh NM-15 was introduced at 3-day intervals. Cultures were maintained in a humidified incubator with 5% CO_2_, 95% air at 37 °C. On day 7 post-plating, after the cells reached confluence, the flasks were shaken overnight in an orbital shaker at 280 rpm at 37 °C to dislodge oligodendrocytes and microglia. Floating cells were removed and the adhering astrocytes were rinsed with NM-15. The cultures were then treated with 100 μM Cytosine-1-β-d-arabinofuranoside (AraC; Sigma-Aldrich) in NM-15 for 3 days. The astrocytes were then detached by trypsinization (0.05% trypsin-ethylenediaminetetraacetic acid (Trypsin-EDTA); Thermo Fisher Scientific), washed twice with NM-15, and re-plated in poly-d-lysine-coated culture flasks in NM-15 at a 1:2 split ratio. Astrocytes were passaged three times as described above within a total period of 17 days and thereafter, they were trypsinized and re-plated in poly-d-lysine coated 24-well plates (Corning, Tewksbury, MA, USA), in NM-15 and incubated overnight. The medium was then gradually replaced with MEM supplemented with 1% FBS and 10 units/ml penicillin/streptomycin. Following overnight incubation in this medium, the astrocyte cultures were used in experiments described below.

To ensure that astrocyte cultures were not contaminated by microglia, cells were fixed in 4% paraformaldehyde in 10 mM phosphate-buffered saline (138 mM NaCl and 2.7 mM KCl), pH 7.4 (PF/PBS), and immunostained with an antibody against glial fibrillary acidic protein (GFAP; Agilent Technologies, Santa Clara, CA, USA), an astrocyte marker, or ionized calcium binding adapter molecule-1 (Iba-1; Wako Laboratory Chemicals, Richmond, CA, USA), a microglial marker, using the corresponding Alexa Fluor 488- or Alexa Fluor 594-tagged secondary antibodies (Thermo Fisher Scientific). The percentage of each cell type was determined by counting ten fields using a 20× objective. The astrocyte cultures were more than 99% pure.

### Preparation of peritoneal cavity cells

Mouse peritoneal cavity cells were isolated from 10-week-old C57BL/6 female mice as described by Ray and Dittel [31]. Briefly, mice were euthanized by exposure to CO_2_. The outer skin of the peritoneum was removed using scissors and forceps, whereas the inner skin of the peritoneum remained intact. Five milliliter of ice-cold sterile phosphate buffer saline (PBS; Thermo Fisher Scientific) containing 3% FBS were injected into the peritoneal cavity. Following a gentle massage of the peritoneum, the peritoneal fluid was collected and centrifuged at 1500 rpm for 8 min. The precipitated cells were suspended in MEM and seeded in 24-well plates for 10 min to allow adherence of macrophages to the dish. MEM was then removed and the cells were incubated in MEM supplemented with 1% FBS and 10 units/ml penicillin/streptomycin. In the experiments that used inserts, the peritoneal cells were seeded and maintained in MEM supplemented with 1% FBS and 10 units/ml penicillin/streptomycin.

### Chemotaxis assay

#### Astrocyte-induced chemotaxis of peritoneal macrophages

Astrocytes, prepared as described above, were seeded into 24-well plates at a density of 1.5 × 10^4^ cells/well in NM-15 and incubated in a humidified incubator overnight. Thereafter, the medium was replaced first with NM-15/MEM containing 1% FBS (1:1, for 8 h) and subsequently, with MEM containing 1% FBS. Following overnight incubation, cells were treated with 1 μM ODN 2088 (InvivoGen, San Diego, CA, USA) or vehicle for 24 h. Transwell inserts, containing the mouse peritoneal cavity cells, were transferred into the 24-well plate that comprised the ODN 2088- or vehicle-treated astrocytes. The co-cultures were incubated for 3 h at 37 °C. This period of time was sufficient for chemotaxis to the lower membrane without cell loss. The inserts were then removed and fixed in 4% PF/PBS. Cells on the upper surface of the inserts were wiped off and the cells that migrated to the lower surface of the inserts were blocked with 30% normal goat serum (NGS; Vector Laboratories, Burlingame, CA, USA) in PBS for 1 h at room temperature (RT). Thereafter, cells were immunostained with F4/80 antibody (Novus Biologicals, Littleton, CO, USA; 1:500 dilution) and the corresponding Alexa Fluor 488-tagged secondary antibody. Samples were counterstained with 4′,6-diamidino-2-phenylindole (DAPI) and coverslipped with ProLong Diamond antifade mountant (Thermo Fisher Scientific). In each experiment, 3-4 replicates/experimental group were used. F4/80^+^ cells in three random and non-overlapping fields of each insert were counted utilizing a Leica DMI3000 fluorescent microscope (Leica Microsystems Inc., Buffalo Grove, IL, USA) equipped with a 20× objective. Representative fluorescent images were captured on a Nikon A1R confocal microscope (Nikon Instruments Inc., Melville, NY, USA) using NIS Elements AR 4.00.07 software (Nikon Instruments Inc.)

#### Astroglial conditioned medium-induced chemotaxis of peritoneal macrophages

Astrocytes were prepared and treated with 1 μM ODN 2088 for 24 h as described above. Thereafter, the astrocyte conditioned medium (CM) was transferred into a new 24-well plate. The Transwell inserts, containing the mouse peritoneal cavity cells, were placed into the 24-well plate and incubated at 37 °C for 3 h. F4/80 immunolabeling of cells and counting were performed as described above for the co-cultures. In some of the experiments, mouse chemokine (C-C motif) ligand 1 (CCL1) neutralizing antibody (1 μg/ml; R&D Systems, Minneapolis, MN, USA), rat IgG_2A_ isotype control (1 μg/ml; R&D Systems), or recombinant chemokines were added to the CM.

### Determination of macrophage polarization, in vitro

Peritoneal cavity cells, prepared as described above, were seeded into 24-well plates at a density of 3.5 × 10^5^ cells/well in MEM and incubated in a humidified incubator for 10 min. Subsequently, the medium containing the non-adhering cells was removed. The adhering macrophages were cultured in MEM containing 1% FBS (control medium), CM of vehicle-treated astrocytes, or CM of ODN 2088-treated astrocytes. In some of the experiments, polarization to the M1 phenotype was induced by adding 20 ng/ml IFN-γ to control medium [32]. Neutralization of CCL1, CCL2, and CCL9 was achieved by adding 1 μg/ml of neutralizing antibodies (R&D Systems). IgG_2A_ (1 μg/ml) was used as isotype control. In experiments investigating the role of CCL9 in macrophage polarization, recombinant mouse CCL9 (rmCCL9; R&D Systems) was used at the final concentration of 20 pg/ml.

After 24-h incubation, macrophage cultures were fixed in 4% PF/PBS and immunostained with an antibody against F4/80 (Novus Biologicals; 1:500), Arginase-1 (Arg-1, MilliporeSigma, Burlington, MA, USA; 1:500), or inducible nitric oxide synthase (iNOS; Cell Signaling Technology, Danvers, MA, USA; 1:500), followed by the corresponding Alexa Fluor 488- or Alexa Fluor 594-tagged secondary antibodies (Thermo Fisher Scientific), and counterstained with DAPI. The percentage of F4/80^+^ macrophages in the cell cultures was determined by counting ten fields per well utilizing a Leica DMI3000 fluorescent microscope equipped with a 40× objective. Representative fluorescent images were captured on a Nikon A1R confocal microscope using NIS Elements AR 4.00.07 software.

### Chemokine array

To identify the chemokines released by ODN 2088- and vehicle-treated astrocytes, the CM was analyzed using the Mouse Chemokine Array C1 (RayBiotech, Norcross, GA, USA) according to the manufacturer’s instructions. The chemiluminescent signal was visualized using a ChemiDoc Touch Imaging System (Bio-Rad, Hercules, CA, USA) and quantified utilizing The Image Lab software (Bio-Rad). The chemokine array enabled the assessment of the following 25 chemokines: CCL1, CCL2, CCL3, CCL5, CCL9, CCL11, CCL12, CCL17, CCL19, CCL20, CCL21, CCL22, CCL24, CCL25, CCL27, chemokine (C-X-C motif) ligand 1 (CXCL1), CXCL2, CXCL4, CXCL5, CXCL9, CXCL11, CXCL12, CXCL13, CXCL16, CX3CL1.

### Enzyme-linked immunosorbent assay

The concentrations of CCL1, CCL2, CCL9, TGF-β, and IL-4 in ODN 2088- or vehicle-treated astrocyte CM were quantified using enzyme-linked immunosorbent assay (ELISA) kits. CCL1, CCL2, and CCL9 ELISA kits were purchased from RayBiotech. TGF-β and IL-4 ELISA kits were obtained from R&D Systems. Recombinant CCL1, CCL2, CCL9, TGF-β, and IL-4 protein standards were dissolved in MEM containing 1% FBS to ensure that CM and protein standards were in the same medium. In addition, CCL1 concentrations were independently measured by a second ELISA kit purchased from a different vendor (Rockland Immunochemicals; Limerick, PA, USA). The colorimetric optical densities (OD) were measured by the Infinite M200 spectrophotometer (Tecan, Morrisville, NC, USA).

### Spinal cord injury and intrathecal delivery of ODN 2088

Nine-week-old female C57BL/6 mice were anesthetized with ketamine (80 mg/kg; Vedco Inc., Saint Joseph, MO, USA) and xylazine (10 mg/kg; Akorn Inc., Lake Forest, IL, USA), administered by intraperitoneal (i.p.) route. A laminectomy was performed at the eighth thoracic vertebral level (T8), and a severe contusion injury (70 kdyne force) was induced using the Infinite Horizon Impactor (Precision Systems & Instrumentation, Lexington, KY, USA). After surgery, the mice were group-housed in cages which were maintained on a heating pad at 37 °C. Immediately following surgery, mice were administered subcutaneously 1 ml of lactated Ringer solution (Baxter, Deerfield, IL, USA), physiologic saline (Baxter), Baytril (0.05 ml; Bayer, Shawnee, KS, USA), and Buprenorphine SR (1 mg/kg; ZooPharm, Laramie, WY, USA). Antibiotics and 1 ml saline were administered twice daily, and analgesics were administered every 3 days for 7 days. Bladders were manually expressed twice a day. Twenty-four hours post-injury (p.i.), open-field locomotor function was assessed using the Basso Mouse Scale (BMS) [33]. The inclusion criterion was a BMS score ≤ 2 on day 1 p.i. This ensured that all subjects sustained a comparable injury. In the present study, all mice sustaining an SCI satisfied the inclusion criterion.

ODN 2088 was dissolved in endotoxin-free distilled water. Intrathecal delivery of ODN 2088 or vehicle started 24 h p.i. and was repeated every 48 h until 14 day p.i. Mice were randomly divided into treatment groups and lumbar punctures (LPs) were performed, under isoflurane (1.0 l/min at a concentration of 3.0% in oxygen) anesthesia. A 27-gauge needle, attached to a Hamilton syringe, was inserted percutaneously into the subarachnoid space between the L5 and L6 laminae. ODN 2088 (150 ng/g body weight) or vehicle was injected in a total volume of 6 μl. The needle was maintained in place for 30 s to prevent egress.

### Immunohistochemistry

Mice were sacrificed on day 14 p.i. by transcardial perfusion with saline followed by 4% PF/PBS. The SCs were dissected, post-fixed in 4% PF/PBS, cryoprotected in 27% sucrose/PBS, embedded in optimal cutting temperature (OCT) compound, frozen in a dry ice-ethanol slurry, and stored at − 80 °C. Thirty-micrometer-thick horizontal cryostat sections were obtained from both groups and thaw-mounted on the same slide.

Spinal cord sections were blocked with 10 mM PBS, pH 7.4 containing 10% NGS and 0.5% Triton X-100, for 1 h at RT. Subsequently, they were incubated overnight at 4 °C with anti-F4/80 (Novus Biologicals; 1:500 dilution), anti-Arg1 (MilliporeSigma; 1:500 dilution), and iNOS (BD Biosciences, San Jose, CA, USA; 1:100 dilution) antibodies. Thereafter, the sections were rinsed with PBS and incubated with the corresponding Alexa Fluor 488- or Alexa Fluor 594-tagged secondary antibodies (Thermo Fisher Scientific) for 1 h at RT. Slides were coverslipped with ProLong Diamond antifade mountant with 4′,6-diamidino-2-phenylindole (DAPI; Thermo Fisher Scientific).

### Stereological quantification of macrophages

A total of four horizontal sections at 150 μm intervals, scanning the injury site in the entire dorso-ventral axis were used. In each section, 500-μm-wide zones, immediately adjacent to the rostral and caudal border of the lesion, were delineated and analyzed. F4/80 and F4/80/Arg-1 immunopositive cell counts were obtained using the Optical Fractionator probe of Stereo Investigator V.11.11.2 (MBF Bioscience, Williston, VT, USA) and an Olympus BX51 microscope (Olympus Inc., Melville, NY, USA) equipped with a 40× objective. The counting frame was 100 μm × 100 μm, the dissector height was 15 μm, and the guard zones were 2 μm. Fifteen percent of the contour was sampled using randomized systematic sampling protocols. In each section, the number of labeled cells was estimated based on planimetric volume calculations in Stereo Investigator. F4/80^+^ and F4/80^+^/Arg-1^+^ cell numbers were estimated and the percentage of F4/80/Arg-1-positive cells was calculated and reported. Representative fluorescent images were captured on a Nikon A1R confocal microscope with a 40× objective using NIS Elements AR 4.00.07 software.

### Statistical analyses

IBM SPSS 20.1 Statistics software (Armonk, NY, USA) and GraphPad Prism 6 software (San Diego, CA, USA) were used for statistical analyses. Two-tailed independent-sample *t*-test and one-way analysis of variance (ANOVA) followed by Tukey’s *post hoc* multiple comparison test were used. A *p* value < 0.05 was considered to be statistically significant. All data are presented as the mean ± standard error of the mean (SEM).

## Results

### TLR9 antagonism significantly increases astrocyte-induced chemotaxis of peritoneal macrophages

**Fig. 1:**
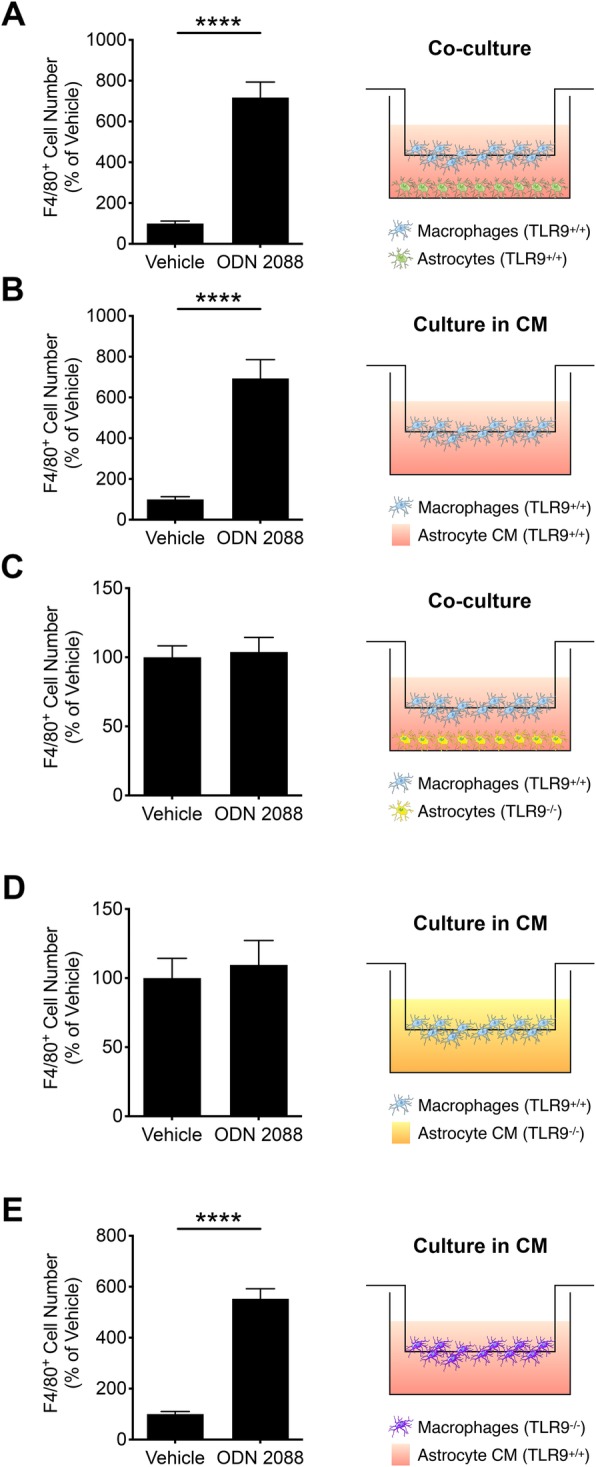
ODN 2088 promotes astrocyte-induced chemotaxis of peritoneal macrophages, in vitro. **a** The number of F4/80^+^ cells that crossed to the lower surface of the membrane in TLR9^+/+^ astrocyte-macrophage co-cultures treated with vehicle or ODN 2088 [****p < 0.0001, independent-sample *t*-test, two-tailed]. **b** The number of F4/80^+^ cells that crossed to the lower surface of the membrane in response to CM derived from vehicle- or ODN 2088-treated TLR9^+/+^ astrocyte cultures [****p < 0.0001, independent-sample *t*-test, two-tailed]. **c** The number of F4/80^+^ cells that crossed to the lower surface of the membrane when TLR9^−/−^ astrocyte were co-cultured with TLR9^+/+^ macrophages and treated with vehicle or ODN 2088 [*p* = 0.7764, independent-sample *t*-test, two-tailed]. **d** The number of F4/80^+^ cells that crossed to the lower surface of the membrane in response to CM derived from vehicle- or ODN 2088-treated TLR9^−/−^ astrocyte cultures [*p* = 0.6776, independent-sample *t*-test, two-tailed]. **e** The number of F4/80^+^ TLR9^−/−^ macrophages that crossed to the lower surface of the membrane in response to CM derived from vehicle- or ODN 2088-treated TLR9^+/+^ astrocyte cultures [****p < 0.0001, independent-sample *t*-test, two-tailed]. The experiments were independently repeated three times, and the mean of three experiments (*n* = 3) is shown. Data are presented as mean ± SEM

To determine whether the TLR9 antagonist ODN 2088 alters the chemoattractant properties of astrocytes, we used a Transwell co-culture system described in the “Methods” section and illustrated in Fig. 1. Astrocytes were cultured in multi-well plates. Peritoneal cavity cells were seeded in Transwell inserts equipped with a porous membrane and the inserts were introduced into the wells containing the astrocytes. In such a co-culture system, peritoneal cells can pass through the pores of the membrane and migrate from the upper surface to the lower surface of the membrane in response to a gradient of chemoattractants released by astrocytes. The number of peritoneal cells that migrate to the lower surface of the insert membrane depends on the potency of the chemoattractant gradient present in the culture wells and can be quantified after visualization of the peritoneal cells by immunocytochemistry using antibodies against specific markers.

We pretreated astrocytes with vehicle and 1 μM ODN 2088 for 24 h. We then introduced the inserts containing peritoneal cells and incubated the co-cultures for 3 h. Subsequently, we fixed and immunolabeled the cells that crossed to the lower surface of the membrane with an antibody against F4/80, a macrophage marker. In co-cultures that contained the vehicle-treated astrocytes, the number of F4/80 immunoreactive cells (F4/80^+^) that migrated to the lower surface of the membrane was low. However, in co-cultures containing the astrocytes that were treated with ODN 2088, the number of F4/80^+^ cells that migrated to the lower surface of the membrane was 7.2-fold higher than those exposed to vehicle-treated astrocytes (Fig. 1a; *p* < 0.0001), suggesting that TLR9 antagonism enhances astrocyte-induced chemotaxis of macrophages.

We next determined whether the effects of ODN 2088 on astrocyte-induced macrophage chemotaxis necessitate the simultaneous presence of astrocytes and macrophages in the Transwell co-cultures and the active cross-talk between the two cell types. To address this question, we treated astrocytes with vehicle or ODN 2088, collected the CM, and exposed the peritoneal cells plated in inserts to astrocyte CM for 3 h. The number of F4/80^+^ cells that crossed to the lower surface of the membrane was 6.9-fold higher when peritoneal cells were exposed to CM of ODN 2088-treated astrocytes compared to CM of vehicle-treated astrocytes (Fig. 1b; *p* < 0.0001). These results suggested that ODN 2088 modulates astrocyte-induced chemotaxis of peritoneal macrophages by modifying the release of chemoattractants by astrocytes through direct actions.

To demonstrate that the effects of ODN 2088 are dependent on the expression of TLR9 in astrocytes, peritoneal cavity cells were either co-cultured with TLR9^−/−^ astrocytes that were pre-treated with ODN 2088 or vehicle (Fig. 1c), or were exposed to CM of ODN 2088- and vehicle-treated TLR9^−/−^ astrocytes, as described above (Fig. 1d). There were no statistical differences in the number of F4/80^+^ cells that migrated to the lower surface of the membrane in the ODN 2088 and vehicle groups, indicating that the differential effects of ODN 2088 on astrocyte-induced chemotaxis of macrophages are dependent on the expression of TLR9 in astrocytes.

Because macrophages express TLR9 in vitro (Additional file 1-A), we undertook experiments to rule out the possibility of direct effects of ODN 2088 on macrophages. Even though astrocyte CM is collected 24 h after addition of ODN 2088 to the cultures, and despite the vulnerability of ODNs to degradation by nucleases released into the medium, we postulated that astrocyte CM could contain *residual* ODN 2088 as the phosphorothioate backbone of the antagonist confers nuclease resistance. Therefore, we ascertained that increased migration is not due to direct antagonism of macrophage TLR9 by *residual* ODN 2088. To address this issue, we first assessed whether ODN 2088 alone, in the absence of astrocytes, affects macrophage chemotaxis. Using the Transwell system described above, we supplemented the medium with ODN 2088 or vehicle and thereafter, we introduced the inserts containing the peritoneal cells into the wells for 3 h. The number of F4/80^+^ cells that migrated from the upper to the lower surface of the insert membrane was not statistically different when cells were exposed to ODN 2088 or vehicle (Additional file 1-B). In addition, we performed experiments similar to those described above by using peritoneal cavity cells isolated from TLR9^−/−^ mice. The results obtained with TLR9^−/−^ peritoneal cells were comparable to those obtained with TLR9^+/+^ cells. The number of F4/80^+^ cells that migrated to the lower surface of the membrane was 5.5-fold higher when TLR9^−/−^ peritoneal cells were exposed to CM of ODN 2088-treated astrocytes compared to CM of vehicle-treated astrocytes (Fig. 1e; *p* < 0.0001). These experiments ruled out the possibility that increased macrophage migration in response to CM of ODN 2088-treated astrocytes is due to the direct effects of the *residual* antagonist on macrophages.

Finally, we assessed whether CM of astrocytes treated with a TLR9 agonist, ODN 1826 (1 μM), would have opposite, inhibitory effects on macrophage migration. We did not find significant differences in the number of F4/80^+^ cells in cultures exposed to CM of vehicle-treated astrocytes and CM of ODN 1826-treated astrocytes (results not shown). However, it is important to emphasize that these studies were limited by the low number of cells that crossed to the lower side of the membrane in both groups, which hampered the detection of an inhibitory effect.

### TLR9 antagonism modulates chemokine release by astrocytes, in vitro

**Fig. 2:**
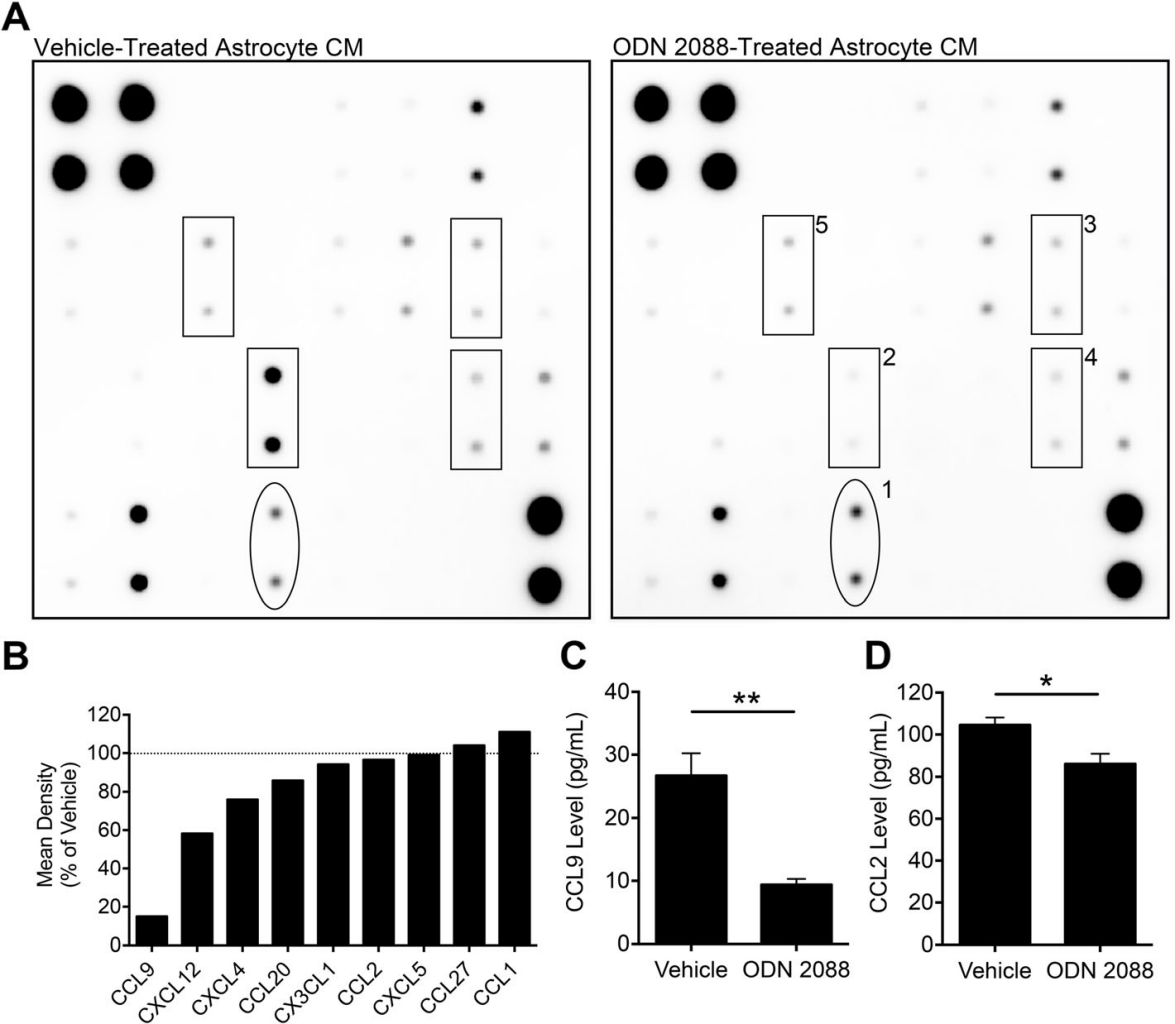
ODN 2088 modulates the release of chemokines by SC astrocytes, in vitro. **a** Representative chemokine arrays used to detect chemokines in CM of vehicle- and ODN 2088-treated astrocytes. The chemokine arrays were independently repeated twice, showing similar results. Results from a representative experiment are shown. The dots enclosed in rectangular boxes show chemokines whose levels were decreased (> 5% difference) in the CM of ODN 2088-treated astrocytes compared to the CM of vehicle-treated astrocytes. The dots enclosed in the oval box show the chemokine whose levels were increased (> 5% difference) in the CM of ODN 2088-treated astrocytes compared to CM of vehicle-treated astrocytes. 1: CCL1; 2: CCL9/MIP-1γ; 3: CCL2/MCP-1; 4: CCL20/MIP-3α; 5: CX3CL1. **b** Densitometric quantification of the signal obtained in the chemokine array using the Image Lab software (Bio-Rad). **c** Quantification of CCL9 levels in CM obtained from ODN 2088- or vehicle-treated astrocytes [**p < 0.01, independent-sample *t*-test, two-tailed]. The experiment was independently repeated four times, and the mean of 4 experiments (*n* = 4) is shown. **d** Quantification of CCL2 levels in CM obtained from ODN 2088- or vehicle-treated astrocytes [*p < 0.05, independent-sample *t*-test, two-tailed]. The experiment was independently repeated three times, and the mean of 3 experiments (*n* = 3) is shown

Since we found that ODN 2088 modifies astrocyte-induced chemotaxis of macrophages through the release of chemoattractants into the CM, we undertook studies to determine which soluble factors could be responsible for this effect. We focused on chemokines since they are potent mediators of chemotaxis [34]. We analyzed the CM of vehicle- or ODN 2088-treated astrocytes by use of a chemokine array that enabled the screening of 25 chemokines as described in the “Methods” section. CCL1 showed the highest increase and CCL9 exhibited the most pronounced decrease in the CM of ODN 2088-treated astrocytes compared to CM of vehicle-treated controls (Fig. 2a). In addition, CCL27 was modestly increased and CCL2, CCL20, CXCL4, CXCL12, and CX3CL1 were decreased, whereas CXCL5 was unaltered (Fig. 2a, b). The signals obtained for the other chemokines were at or below detection limit and could not be reliably quantified.

To further corroborate the results obtained by chemokine arrays, we used ELISA to quantify CCL1, CCL2, and CCL9. In agreement with the arrays, CCL9 concentrations in the CM of ODN 2088-treated astrocytes were significantly reduced by 65% compared to CCL9 concentrations in the CM of vehicle-treated controls (Fig. 2c, *p* < 0.01). Similarly, CCL2 concentrations were decreased by 11% in the CM of ODN 2088-treated cultures compared to control CM (Fig. 2d, *p* < 0.05). However, CCL1 levels could not be quantified by ELISA even though we used kits obtained from two different vendors and assessed several different CM preparations, which were successfully utilized to quantify CCL2 and CCL9 levels. This discrepancy could be due to differences in the sensitivity of the chemokine array versus ELISA, since in the chemokine array the signal is amplified by use of a chemiluminescent substrate.

To confirm the involvement of TLR9 in the regulation of chemokine release by astrocytes, we treated the cultures with the TLR9 agonist (ODN 1826) and assessed CCL2 and CCL9 levels in the CM by ELISA. We anticipated an opposite effect to that obtained by use of the antagonist and therefore, we expected an increase in the release of CCL2 and CCL9. As anticipated, ODN 1826 increased CCL2 and CCL9 release by 5.6-fold and 1.9-fold respectively compared to vehicle-treated astrocyte CM (Additional file 2). In the case of CCL1, we anticipated a decrease in CCL1 levels in response to ODN 1826. However, as mentioned above, CCL1 levels were below the detection limit of the ELISA and could not be quantified in any of the groups.

### CCL1 released by ODN 2088-treated astrocytes mediates the chemotaxis of peritoneal macrophages

**Fig. 3:**
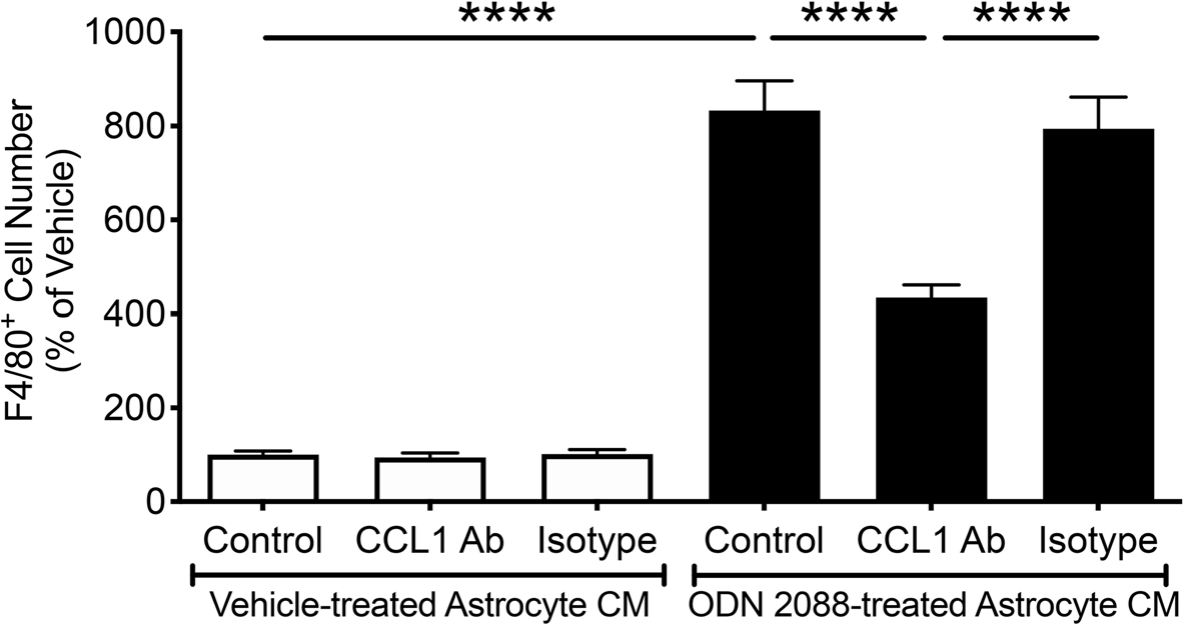
CCL1 released by ODN 2088-treated astrocytes mediates the chemotaxis of peritoneal macrophages. Quantification of F4/80^+^ cells that crossed to the lower surface of the membrane in response to CM derived from vehicle- or ODN 2088-treated TLR9 astrocytes in the absence or presence of CCL1 neutralizing antibody or IgG_2A_ isotype control [*F* (5, 48) = 81.03, *p* < 0.0001 by one-way ANOVA, ****p < 0.0001 by Tukey’s *post hoc* test]. The results of three independent experiments (*n* = 3) are shown. Data are presented as mean ± SEM

The chemokine array identified CCL1 as the principal chemokine whose release was increased in response to treatment of astrocytes with ODN 2088. Therefore, we investigated whether CCL1 could be a trigger that elicits macrophage migration by ODN 2088-treated astrocytes or their CM. We addressed this question by using neutralizing antibodies to block CCL1 in astrocyte CM. Peritoneal cavity cells, seeded in Transwell inserts, were cultured with CM obtained from ODN 2088- or vehicle-treated astrocytes in the presence or absence of a CCL1 neutralizing antibody. Vehicle or IgG_2A_ isotype were used as controls. The number of F4/80^+^ cells that migrated to the lower surface of the membrane was 8.3-fold higher when peritoneal cells were exposed to CM of ODN 2088-treated astrocytes compared to CM of vehicle-treated astrocytes (Fig. 3; *p* < 0.0001). When CCL1 neutralizing antibody was introduced into the cultures containing the CM of ODN 2088-treated astrocytes, the number of F4/80^+^ cells that crossed to the lower surface of the membrane was significantly reduced by 47.84% (Fig. 3; *p* < 0.0001). Addition of IgG_2A_ isotype did not reduce the number of F4/80^+^ immunoreactive cells that migrated to the lower surface of the membrane, confirming the specificity of the effect exerted by the neutralizing antibody (Fig. 3). These results indicated that CCL1 released by ODN 2088-treated astrocytes mediates, at least partly, the increase in macrophage chemotaxis.

We performed additional experiments to further corroborate the role of CCL1 in astrocyte-induced chemotaxis of macrophages. We exposed macrophages plated in inserts to control medium (MEM containing 1% FBS) or vehicle-treated astrocyte CM, with and without addition of recombinant mouse CCL1 (rmCCL1; 100 pg/ml) and evaluated macrophage migration. In both experiments, the number of F4/80^+^ cells that crossed to the lower side of the membrane was increased in cultures containing rmCCL1 (Additional file 3). These studies further confirm that CCL1 mediates the chemotactic migration of macrophages.

### Effectors released by ODN 2088-treated astrocytes modulate macrophage phenotype

**Fig. 4:**
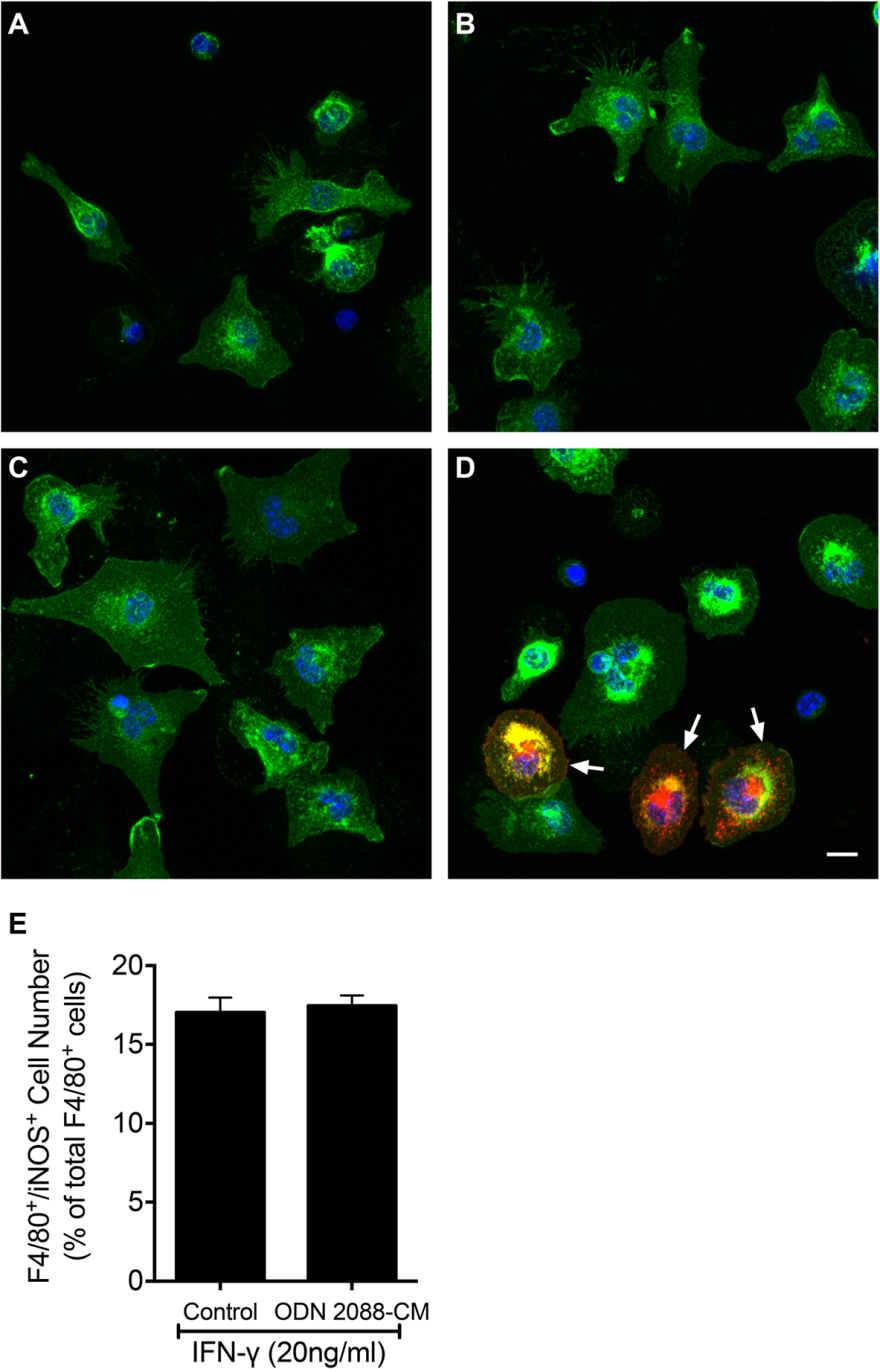
CM of ODN 2088-treated astrocytes does not induce M1 macrophage polarization, in vitro. **a–d** Representative fluorescent images showing mouse peritoneal macrophages treated with **a** control medium (MEM containing 1% FBS), **b** CM of vehicle-treated astrocytes, **c** CM of ODN 2088-treated astrocytes, or **d** 20 ng/ml IFN-γ in control medium. The cells were double-labeled with F4/80 (green) and iNOS (red), and counter stained with DAPI (blue). F4/80^+^/iNOS^+^ double-labeled cells (arrows) were only present in macrophage cultures treated with IFN-γ. **e** Addition of CM of ODN 2088-treated astrocytes (2088-CM) does not alter the number of F4/80^+^/iNOS^+^ cells in IFN-γ-treated macrophage cultures [*p* = 0.7344, independent-sample *t*-test, two-tailed]. The experiment was independently repeated twice, yielding similar results. Results from a representative experiment are shown. Data from the independent biological repeat of these experiments can be found in Additional file 8A. Data are presented as mean ± SEM

We performed experiments to determine whether SC astrocytes modulate the polarization of macrophages into the M1 or M2 phenotype and if this capacity is altered by ODN 2088 treatment. We first focused on M1 macrophages which were identified by F4/80^+^/iNOS^+^ double-labeling using immunocytochemistry [35]. When peritoneal cavity cells were cultured in control medium (MEM containing 1% FBS), about 88% of the cells were F4/80^+^, indicating that the majority of the peritoneal cells in our cultures are macrophages. These cells were not immunoreactive for iNOS (Fig. 4a). Exposure to vehicle- or ODN 2088-treated astrocyte CM for 24 h did not induce expression of iNOS in F4/80^+^ cells (Fig. 4b, c) demonstrating that factors secreted by astrocytes do not promote M1 polarization. In contrast, macrophages treated with IFN-γ, a trigger that promotes M1 polarization, expressed iNOS (Fig. 4d), indicating that the macrophages conserve the capacity to acquire the M1 phenotype if presented with the appropriate stimulus. In addition, we assessed whether ODN 2088-treated astrocyte CM counteracts IFN-γ-induced M1 polarization and did not find any significant effects (Fig. 4e). Finally, direct antagonism of macrophage TLR9 by addition of ODN 2088 to peritoneal cell cultures did not alter IFN-γ-induced M1 polarization (Additional file 4A).

**Fig. 5:**
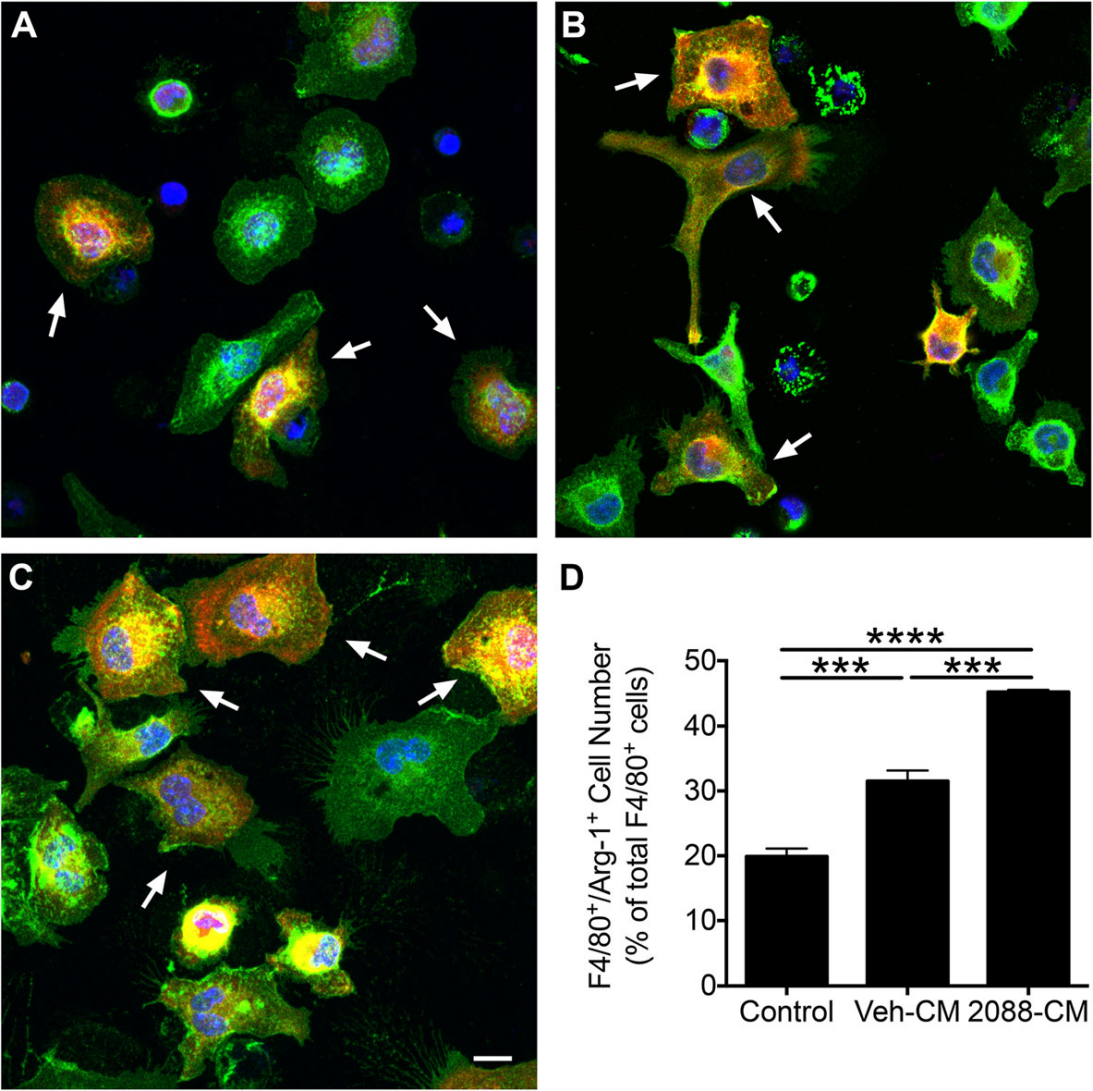
CM of ODN 2088-treated astrocytes regulates M2 macrophage polarization, in vitro. **a**–**c** Representative fluorescent images showing mouse peritoneal macrophages treated with **a** control medium (MEM containing 1% FBS), **b** CM of vehicle-treated astrocytes, or **c** CM of ODN 2088-treated astrocytes. The cells were double-labeled with antibodies against F4/80 (green) and Arg-1 (red), and counter stained with DAPI (blue). Arrows point at examples of F4/80^+^/Arg-1^+^ double-labeled cells. Scale bar: 10 μm. **d** Quantification of F4/80^+^/Arg-1^+^ cells in the macrophage cultures maintained in control medium and in CM of vehicle- (Veh-CM) or ODN 2088 (2088-CM)-treated astrocytes [*F* (2, 6) = 120.6, *p* < 0.0001 by one-way ANOVA, ***p < 0.001, ****p < 0.0001 by Tukey’s *post hoc* test]. The experiment was independently repeated three times, yielding similar results. Results from a representative experiment are shown. The results obtained in additional biological repeats of these experiments can be found in Additional file 8B and C. Data are presented as mean ± SEM

Subsequently, we determined whether the F4/80^+^ cells express Arg-1, a marker for M2 macrophages [36]. In control medium, some of the F4/80^+^ cells were immunoreactive for Arg-1 (Fig. 5a). The percentage of F4/80^+^/Arg-1^+^ cells ranged from 10 to 21% in independent macrophage preparations. These results indicated that the majority of the F4/80^+^ cells maintained in control medium are unpolarized (M0) macrophages, but a subpopulation shows the characteristics of the M2 phenotype. Exposure of macrophage cultures to CM from vehicle-treated astrocytes significantly increased the percentage of F4/80^+^/Arg-1^+^ cells (Fig. 5b and d, *p* < 0.001) albeit this effect was not consistently observed in all the experiments. However, when cells were cultured in the presence of ODN 2088-treated astrocyte CM for 24 h, the percentage of F4/80^+^/Arg-1^+^ cells was significantly increased as compared to cells that were exposed to CM of vehicle-treated astrocytes, and this effect was consistently observed in independent experiments (Fig. 5c, d; *p* < 0.001). Direct blockade of macrophage TLR9 by addition of ODN 2088 to macrophage cultures maintained in control medium or CM of vehicle-treated astrocytes did not alter the percentage of F4/80^+^/Arg-1^+^ cells (Additional file 4B and C) indicating that macrophage TLR9 antagonism does not impact M2 polarization. These results suggest that ODN 2088 enhances the capacity of astrocytes to polarize macrophages into the M2 phenotype.

To corroborate these findings, we also measured expression of IL-10 in macrophage cultures exposed to CM of vehicle- or ODN 2088 astrocytes for 24 h. IL-10 is highly expressed in M2 macrophages [37]. IL-10 transcript levels were significantly increased by 50.03 ± 12.89% (*n* = 5; *p* < 0.05) in macrophages treated with CM of ODN 2088-treated compared to controls. These findings further support the idea that signals derived by ODN 2088-treated astrocytes polarize macrophages into M2 phenotype.

**Fig. 6:**
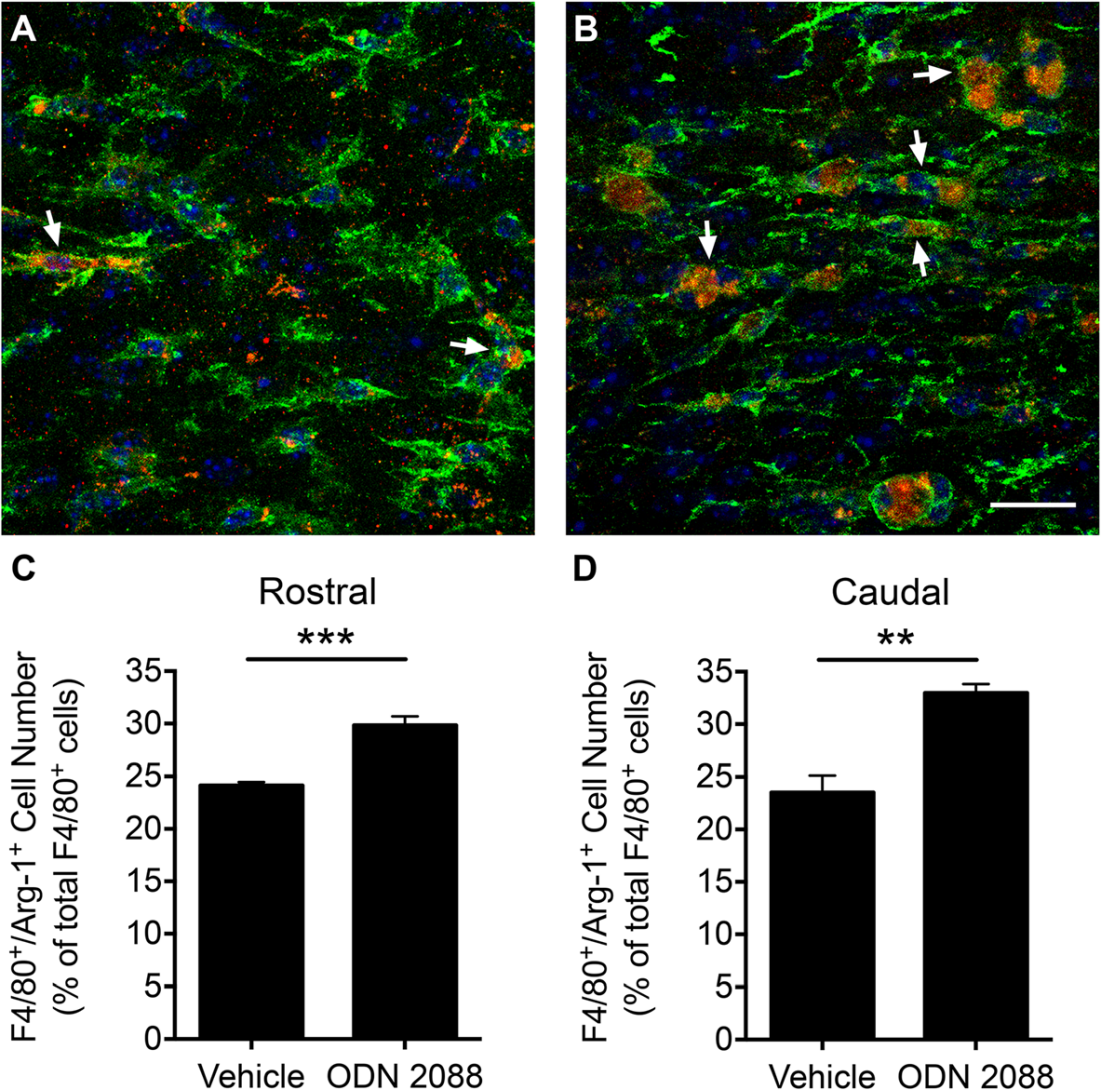
Intrathecal ODN 2088 treatment increases the percentage of F4/80^+^/Arg-1^+^ double-labeled cells at the glial scar following SCI. Representative fluorescent images showing horizontal SC sections of mice that sustained a SCI and treated with **a** vehicle and **b** ODN 2088. The images were obtained from the glial scar. The sections were double-labeled with an antibody against F4/80 (green) and Arg-1 (red), and counter stained with DAPI (blue). Arrows point at examples of F4/80^+^/Arg-1^+^ double-labeled cells. Scale bar: 20 μm. **c** Percentage of F4/80^+^/Arg-1^+^ double-labeled cells within the 500-μm-wide zone immediately adjacent to the rostral border of the lesion core [***p < 0.001, independent-sample *t*-test, two-tailed]. **d** Percentage of F4/80^+^/Arg-1^+^ double-labeled cells within the 500-μm-wide zone immediately adjacent to the rostral border of the lesion core [**p < 0.01, independent-sample *t*-test, two-tailed]. Data are presented as mean ± SEM. *n* = 4 mice/group

Next, we undertook experiments to determine whether ODN 2088 influences the macrophage phenotype in vivo. We used a mouse model of SC contusion injury, which creates an inflammatory milieu at the affected site with extensive macrophage infiltration. We treated mice with ODN 2088 or vehicle until day 14 p.i., the time when a mature glial scar forms around the lesion core and we determined whether F4/80^+^ cells at the injury epicenter are iNOS or Arg-1 immunoreactive. There was a difference in the distribution of F4/80^+^/iNOS^+^ and F4/80^+^/Arg-1^+^ doubled-labeled cells at the lesion core and at the glial scar, which was defined as 500-μm-wide zones immediately caudal or rostral to the border of the lesion core (Additional file 5A) [30]. Within the lesion core, large, hypertrophic F4/80^+^ cells formed a dense network of juxtaposed cells. Both F4/80^+^/iNOS^+^ and F4/80^+^/Arg-1^+^ double-labeled cells could be detected at the lesion core (Additional file 5B and C). In contrast, F4/80^+^ cells were scattered throughout the glial scar and some of these cells were also Arg-1 immunoreactive (Fig. 6a, b). However, we could not detect F4/80^+^/iNOS^+^ double-labeled cells at the glial scar (Additional file 5D). We quantified total F4/80^+^ and F4/80^+^/Arg-1^+^ immunoreactive cells at the glial scar rostral and caudal to the lesion core and calculated the percentage of F4/80^+^/Arg-1^+^ immunoreactive cells in both ODN 2088-treated injured mice and vehicle-treated injured controls. Overall, there was a 24.90 ± 2.66% (*p* < 0.01) decrease in total F4/80^+^ cell number in ODN 2088-treated mice compared to controls. This finding was in conceptual agreement with our earlier investigations showing a decrease in the number of inflammatory cells at the injury epicenter following ODN 2088 treatment [28]. However, the percentage of F4/80^+^/Arg-1^+^ double-labeled cells was significantly increased following ODN 2088 treatment: in the rostral glial scar zone of the vehicle-treated injured mice, 24% of the F4/80^+^ cells were also Arg-1^+^. The percentage of F4/80^+^/Arg-1^+^ cells increased significantly to 30% in ODN 2088-treated mice (Fig. 6c, *p* < 0.001). Similarly, in the caudal glial scar zone of the vehicle-treated injured mice, 24% of the F4/80^+^ cells were also Arg-1^+^. There was a significant increase in the percentage of F4/80^+^/Arg-1^+^ cells to 33% following ODN 2088 treatment (Fig. 6d, *p* < 0.01). Thus, ODN 2088 increases the proportion of M2 cells at the glial scar following SCI.

### Astrocyte-derived CCL2 and CCL9 are negative regulators of macrophage M2 polarization

Cytokines and chemokines are the principal stimuli that drive the polarization of macrophages [38, 39]. TGF-β1 is one of the major cytokines secreted by astrocytes [40] and has been shown to drive M2 polarization [41]. We first postulated that an increase in TGF-β1 in the CM of ODN 2088-treated astrocytes could be the stimulus that enhances the M2 phenotype in our macrophage cultures. We quantified TGF-β1 levels in the CM of vehicle- or ODN 2088-treated astrocytes by ELISA. In contrast to our postulate, TGF-β1 concentrations in the CM of ODN 2088-treated astrocytes were significantly reduced by 17% compared to those measured in the CM of vehicle-treated controls (Additional file 6). This finding ruled out TGF-β1 as the potential effector that enhances the M2 phenotype when macrophages are exposed to the CM of ODN 2088-treated astrocytes. IL-4 is another cytokine that polarizes macrophages into the M2 phenotype [42]. However, IL-4 is not secreted by astrocytes [43], but is released by microglia [44]. Even though our astrocyte cultures contain < 1% contaminating microglia, we performed experiments to assess whether these few microglia release enough IL-4 that drives macrophages into M2 phenotype. However, IL-4 concentrations in both vehicle- and ODN 2088-treated astrocyte CM were below the detection limit of the ELISA. Therefore, we could not establish IL-4 as the stimulus that drives the M2 phenotype in response to CM of ODN 2088-treated astrocyte cultures.

**Fig. 7:**
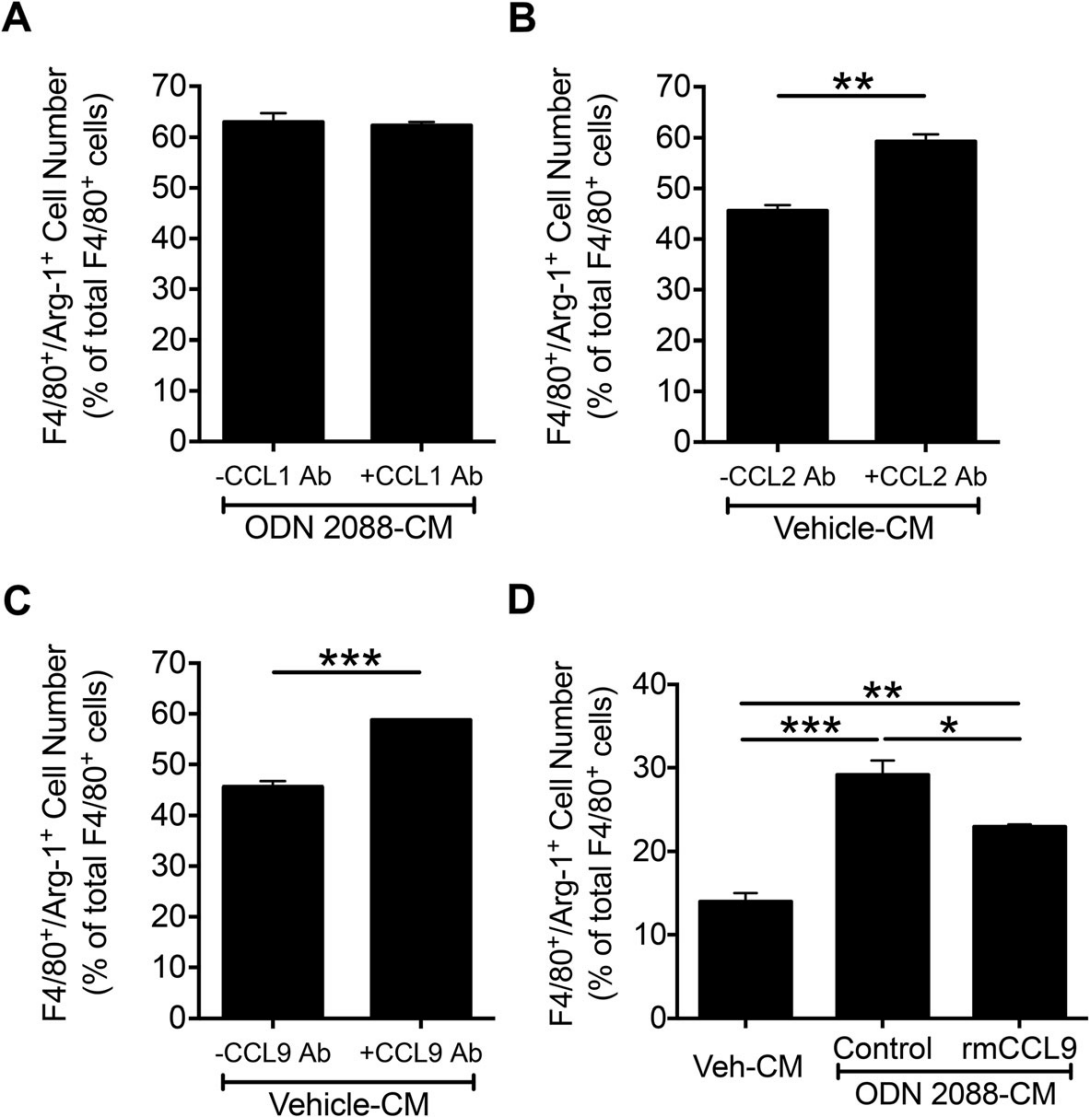
Astrocyte-derived CCL2 and CCL9 but not CCL1 regulate macrophage polarization, in vitro. **a** Macrophage cultures were exposed to ODN 2088-treated astrocyte CM (ODN 2088-CM), in the absence or presence of CCL1 neutralizing Ab. The graph shows the quantification of the F4/80^+^/Arg-1^+^ cell number expressed as percentage of total F4/80^+^ cells in the macrophage cultures [*p* = 0.7228, independent-sample *t*-test, two-tailed]. **b** Macrophage cultures were exposed to vehicle-treated astrocyte CM, in the absence or presence of CCL2 neutralizing Ab. The graph shows the quantification of the F4/80^+^/Arg-1^+^ cell number expressed as percentage of total F4/80^+^ cells in the macrophage cultures [**p < 0.01, independent-sample *t*-test, two-tailed]. **c** Macrophage cultures were exposed to vehicle-treated astrocyte CM, in the absence or presence of CCL9 neutralizing Ab. The graph shows the quantification of the F4/80^+^/Arg-1^+^ cell number expressed as percentage of total F4/80^+^ cells in the macrophage cultures [***p < 0.001, independent-sample *t*-test, two-tailed]. **d** Macrophage cultures were exposed to vehicle-treated astrocyte CM (Veh-CM) or ODN 2088-treated astrocyte CM (ODN 2088-CM) for 24 h, with or without (control) addition of rmCCL9 (20 pg/ml). The graph shows the quantification of the F4/80^+^/Arg-1^+^ double-labeled cells expressed as percent of total F4/80^+^ cells in macrophage cultures [*F* (2, 6) = 53.68, *p* < 0.0001 by one-way ANOVA, *p < 0.05, **p < 0.01, ***p < 0.001 by Tukey’s *post hoc* test]. The experiments were independently repeated twice, yielding similar results. Results from a representative experiment are shown. Results obtained from additional biological repeats of these experiments can be found in Additional file 8D-G. Data are presented as mean ± SEM

Subsequently, we focused on chemokines. It has previously been reported that CCL1 is required for the maintenance of the M2b phenotype [45]. As mentioned above, our chemokine arrays indicated that CCL1 levels are higher in the CM of ODN 2088-treated astrocytes than the CM of vehicle-treated astrocytes. Hence, we investigated whether CCL1 is the effector that promotes the M2 phenotype. We hypothesized that neutralization of CCL1 in the CM of ODN 2088-treated astrocytes by use of antibodies could prevent the increased polarization of the macrophages into the M2 phenotype. However, addition of CCL1 neutralizing antibodies to macrophage cultures exposed to the CM of ODN 2088-treated astrocytes did not alter the percentage of F4/80^+^/Arg-1^+^ cells, indicating that CCL1 is not the mediator of M2 polarization in our cultures (Fig. 7a).

These findings led us to investigate the involvement of CCL2 because a decrease in CCL2 has previously been associated with the development of the M2 phenotype [46]. As mentioned above, cytokine arrays and ELISA showed that ODN 2088 treatment reduces CCL2 release by astrocytes. To establish a link between a decrease in CCL2 and enhancement of M2 phenotype in our macrophage cultures, we added CCL2 neutralizing antibodies to vehicle-treated astrocyte CM and assessed whether blocking CCL2 increases the percentage of F4/80^+^/Arg-1^+^ cells. In fact, neutralization of CCL2 significantly increased the percentage of F4/80^+^/Arg-1^+^ cells (Fig. 7b; *p* < 0.01). These results indicated that astrocyte-derived CCL2 is a negative regulator of M2 polarization and a reduction in CCL2 release by astrocytes in response to ODN 2088 fosters the development of the M2 phenotype.

We performed similar experiments with CCL9, since it was the cytokine that showed the most profound decrease in the CM of ODN 2088-treated astrocytes. When CCL9 neutralizing antibodies were added to macrophage cultures maintained in CM of vehicle-treated astrocytes, the percentage of F4/80^+^/Arg-1^+^ cells was significantly increased (Fig. 7c; *p* < 0.001). Addition of IgG_2A_ isotype to either vehicle- or ODN 2088-treated astrocyte CM did not alter the percentage of F4/80^+^/Arg-1^+^ cells, confirming the specificity of the neutralizing antibodies (Additional file 7). These results indicated that CCL9 is another negative regulator of M2 polarization and ODN 2088 fosters the differentiation of macrophages into the M2 phenotype by reducing CCL9 release by astrocytes. To further corroborate this idea, we restored CCL9 levels in the CM of ODN 2088-treated astrocytes by addition of recombinant mouse CCL9 (rmCCL9). Supplementation of CM of ODN 2088-treated astrocytes with rmCCL9 significantly decreased the percentage of F4/80^+^/Arg-1^+^ cells in macrophage cultures (Fig. 7d; *p* < 0.05).

## Discussion

**Fig. 8:**
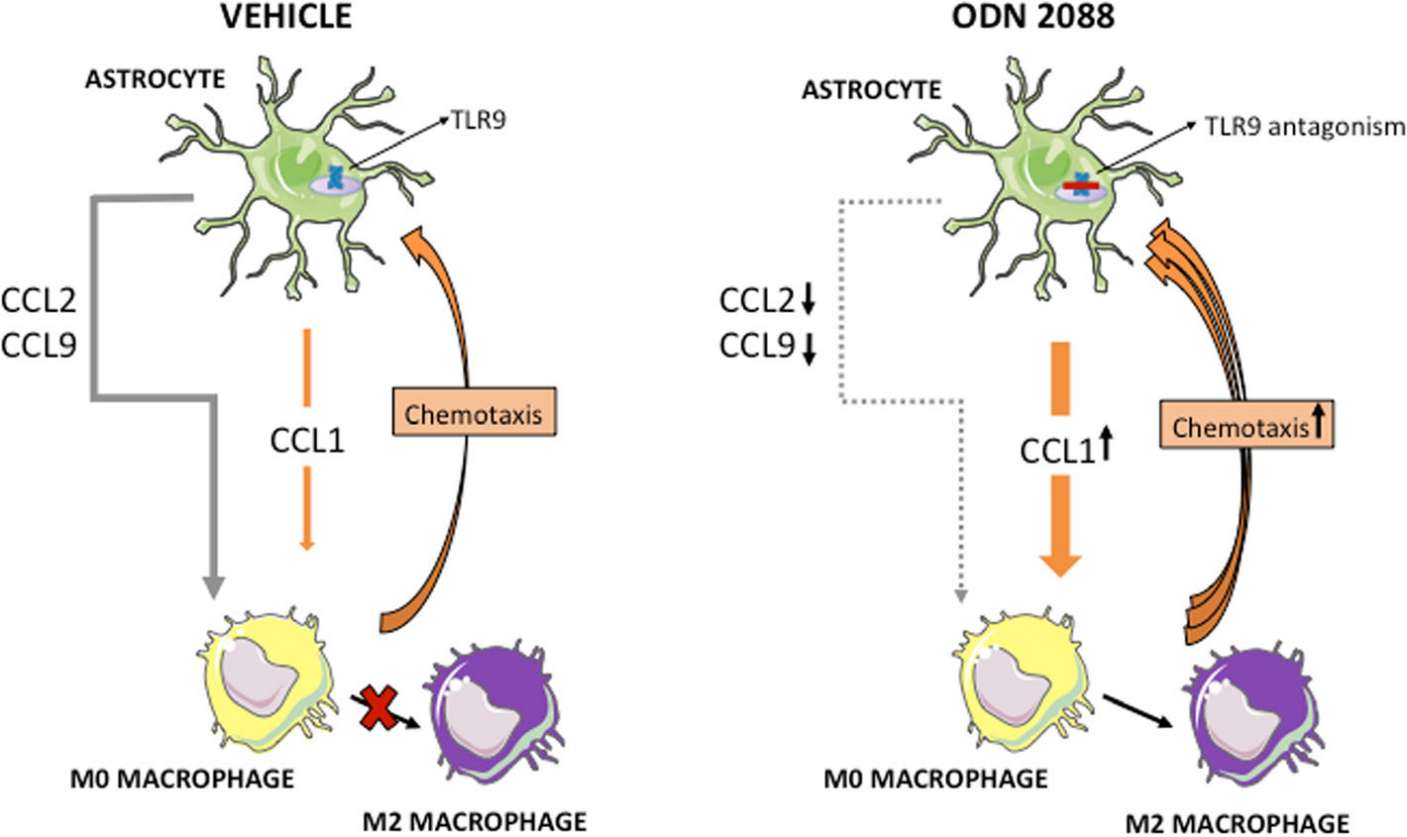
A scheme summarizing the effects of ODN 2088-treated astrocytes on macrophages. TLR9 antagonism increases the release of CCL1 by astrocytes, which enhances macrophage chemotaxis. In contrast, CCL2 and CCL9 release are decreased in response to ODN 2088. This reduces the negative regulatory effect of CCL2 and CCL9 on M2 macrophage polarization and fosters the M2 phenotype

The present investigations show, for the first time, that direct antagonism of TLR9 in SC astrocytes modulates astrocyte-to-macrophage signals and this, in turn, promotes macrophage chemotaxis and polarization into the anti-inflammatory M2 phenotype, in vitro. The TLR9 antagonist modifies the pattern of chemokine release by astrocytes: increased CCL1 release supports macrophage chemotaxis, whereas decreased CCL2 and CCL9 release drives the polarization of macrophages into the M2 phenotype, suggesting that astrocyte-derived CCL2 and CCL9 are negative regulators of M2 polarization (summarized in Fig. 8). Importantly, administration of intrathecal ODN 2088 to mice sustaining a mid-thoracic SC contusion injury increases the percentage of macrophages that express a marker of the M2 phenotype at the glial scar. This finding is consistent with our earlier report showing that TLR9 antagonist modifies the milieu of the glial scar to foster neuroprotection [30].

### Astrocyte-induced chemotaxis of immune cells in CNS pathology: role of chemokines

Several lines of evidence indicate that glial cells are the major source of chemokines in the CNS [47–49]. Astrocytes are among glial subtypes that release chemokines, especially in response to stimulation by cytokines [48, 50, 51]. Increased expression of chemokines in microglia and astrocytes has been associated with the migration of leukocytes into the denervated hippocampus following axotomy [49]. It has been reported that infiltration of T lymphocytes into the brain of patients with glioma coincides with CCL2 expression in GFAP^+^ astrocytes [52]. The same investigators also showed that stereotactic injection of adenoviral vectors into the cerebral cortex of monkeys is paralleled by infiltration of lymphocytes into the injection site and increased CCL2 expression in GFAP^+^ cells [52]. Cytokine-stimulated astrocytes produce chemokines that attract dendritic cells in vitro and chemokine-expressing reactive astrocytes are localized to multiple sclerosis lesions [53]. IL-9-stimulated astrocytes release CCL20 which induces Th17 cell migration, in vitro [54]. Collectively, these reports highlight the key role played by astrocytes in promoting the chemotaxis of immune cells and their infiltration into the CNS in pathological conditions.

### CCL1-mediated chemotaxis of macrophages

Recruitment of monocyte-derived macrophages into the CNS plays an important role in pathological conditions including SCI [55], multiple sclerosis, and amyotrophic lateral sclerosis [56]. In our studies, we identified CCL1 as the major chemoattractant released by ODN 2088-treated astrocytes that mediates macrophage chemotaxis, in vitro. In agreement with our findings, it has been reported that CCL1 is a potent chemoattractant for macrophages [57] and drives the chemotaxis of macrophage colony stimulating factor (M-CSF)-derived human macrophages [58] which exhibit M2 phenotype [59]. Even though CCL1 is a chemokine that is secreted primarily by T cells, monocytes, and mast cells [60], it is also produced by brain astrocytes and mediates the chemotaxis of T regulatory cells during stroke [61]. CCL1 binds its cognate receptor CCR8 which is expressed in peritoneal macrophages and modulates macrophage function including secretion of cytokines in response to stimulation of TLRs [62] and aggregation of macrophages [63].

In contrast to CCL1, CCL2 release by astrocytes was decreased in response to the TLR9 antagonist. This was an intriguing finding, especially because CCL2 is an important chemoattractant for immune cells [54]. The differential release of CCL1 and CCL2 by astrocytes in response to ODN 2088 supports the notion that TLR9 orchestrates astrocyte-to-macrophage signaling such that different macrophage functions are modulated in a specific and selective manner: increased CCL1 release promotes chemotaxis and decreased CCL2 release enables the differentiation of macrophages into the M2 phenotype. It is also important to emphasize that CCL2 plays additional roles in the CNS and has been implicated in the regulation of synaptic function [64]. It is therefore possible that astroglial TLR9 orchestrates the communication of astrocytes with other cells such as neurons through selective changes in the release of astrocyte-derived CCL2.

### Polarization of macrophages: role of chemokines

Macrophages are heterogeneous cells that show considerable functional and phenotypic plasticity [65]. They respond to stimuli in their microenvironment by acquiring distinct phenotypes which are characterized by the expression of specific cell surface molecules, transcriptome and secretome profiles, and effector function. Conventionally, macrophages have been divided into classically activated M1 macrophages and alternatively activated M2 macrophages. M1 macrophages secrete pro-inflammatory cytokines, whereas M2 macrophages are characterized by immunoregulatory properties, low pro-inflammatory cytokine production, and secretion of anti-inflammatory cytokines. M2 macrophages are further classified into M2a, M2b, M2c, and M2d subtypes with distinct properties [6, 37, 66, 67].

The polarization of macrophages into M1 versus M2 phenotypes is primarily driven by cytokines. Th1 cytokines including IFN-γ and tumor necrosis factor-α (TNF-α) induce M1 phenotype, whereas Th2 cytokines including IL-4, IL-10, and IL-13 are essential triggers that promote the M2 phenotype [65]. TGF-β1 has also been shown to foster the M2 phenotype [41]. However, chemokines and their receptors also play a role in the polarization of macrophages [38], even though further elucidation of this role is needed. C-C chemokine receptor type 5 (CCR5) is involved in the modulation of M1 versus M2 macrophages that infiltrate the adipose tissue during obesity-induced inflammation [68]. The role of CCL2 in macrophage polarization has been controversial [45, 68–70]. Treatment of bone marrow-derived macrophages with CCL2 did not polarize the cells into M1 or M2 phenotype but enhanced the response to IFN-γ/lipopolysaccharide (LPS), stimuli that drive M1 polarization. High CCL2 doses suppressed the expression of Arg-1, which is a marker of M2 macrophages [70]. This latter finding is in conceptual agreement with our results suggesting that CCL2 could be a negative regulator of Arg-1 expression and M2 polarization and that a reduction in astrocyte-derived CCL2 secretion in response to ODN 2088 treatment facilitates Arg-1 expression. A link between CCL2 deficiency and increased expression of genes that code for M2 markers, including Arg-1, has also been reported in transgenic mice [46], whereas abrogation of CCL2 fostered M2 polarization and hindered the M1 polarization of adipose tissue macrophages [71]. These studies further support the notion that CCL2 is a negative regulator of M2 phenotype.

Interestingly, we also found that CCL9 could be an additional negative regulator of Arg-1 expression and M2 phenotype. To our knowledge, CCL9 release by SC astrocytes has not been reported before. Although macrophages express CCR1, the CCL9 receptor [72], the contribution of CCL9 to macrophage polarization has not been adequately defined and warrants further investigations.

### Increased M2 macrophages in response to ODN 2088 treatment of mice sustaining a SCI

Our studies show that the majority of F4/80^+^ cells at the glial scar are unpolarized (M0) macrophages that express neither iNOS nor Arg-1 in both vehicle- and ODN 2088-treated injured mice. Nevertheless, intrathecal ODN 2088 significantly increased the percentage of F4/80^+^/Arg-1^+^ double-labeled macrophages at the glial scar. This finding is in conceptual agreement with our in vitro data showing that ODN 2088 influences the polarization of F4/80^+^ macrophages into M2 phenotype via modulation of astrocyte-to-macrophage signals. However, it remains to be determined whether astrocytes are the main cell type that drives M2 polarization in ODN 2088-treated injured mice. Driving macrophages into a phenotype that is more favorable for neuroprotection and repair was associated with improvement of functional deficits following SCI [73, 74].

The mechanisms by which ODN 2088-treated astrocytes elicit macrophage chemotaxis and polarization in vitro require further investigations. In addition to secretion of soluble molecules, extracellular vesicles (EVs) [75] shed by astrocytes could play a role in astrocyte-to-macrophage signaling. Astrocytes shed EVs constitutively, but activation of astrocytes under pathological conditions increases the shedding of astrocyte-derived EVs [76, 77]. The cargo carried by EVs includes cytokines and chemokines [78] and is variable depending on the stimulus that induces the shedding [77]. EVs have been implicated in macrophage chemotaxis [79] and macrophage polarization [80]. Following brain injury, astrocyte-derived EVs mediate the recruitment of peripheral leukocytes into the affected site [81], whereas mesenchymal stem cell-derived EVs promote macrophage polarization into anti-inflammatory phenotype [80]. It is possible that such mechanisms become more effective when astrocyte TLR9 signaling is blocked by ODN 2088 leading to alterations in astrocyte-macrophage communication, affecting chemotaxis and polarization of macrophages.

## Conclusions

We previously reported that TLR9 antagonism modulates astrocyte proliferation, migration, cytokine release, and astrocyte-neuron interactions, in vitro [15, 30]. The present study expands these findings and shows that astrocyte chemoattractant properties and astrocyte-to-macrophage signaling are also modulated by TLR9 antagonism. Importantly, the investigations on mice sustaining a SCI indicate that treatment with ODN 2088 fosters M2 macrophages at the glial scar. This finding complements our earlier report demonstrating alterations in the glial scar in response to ODN 2088 [30, 82]. It remains to be determined whether the in vivo effects of ODN 2088 on macrophage polarization are the outcome of direct inhibition of TLR9 in astrocytes or mediated through effects on other cells such as neurons, microglia, and infiltrating immune cells, which also express TLR9. Our findings could have relevance for a broad range of CNS pathologies in which astrocytes and macrophages play a key role [83].

## Supplementary information

**Additional file 1. **ODN 2088 does not induce chemotaxis of peritoneal macrophages in the absence of astrocytes. (A) TLR9 expression in macrophages. A gel showing TLR9 transcripts in mouse peritoneal cell cultures by qRT-PCR. (B) The number of F4/80^+^ cells that crossed to the lower surface of the insert membrane when peritoneal cells were exposed to ODN 2088 in the absence of astrocytes or astrocyte CM [*p* = 0.2631, independent-sample *t*-test, two-tailed]. The experiment was independently repeated six times, and the mean of six experiments (*n* = 6) is shown. Data are presented as mean ± SEM.

**Additional file 2. **ODN 1826 increases CCL2 and CCL9 release by astrocytes, in vitro. (A) Quantification of CCL2 levels in CM obtained from vehicle- or ODN 1826-treated astrocytes [**p < 0.01, independent-sample *t*-test, two-tailed]. (B) Quantification of CCL9 levels in CM obtained from vehicle- or ODN 1826-treated astrocytes [***p < 0.001, independent-sample *t*-test, two-tailed]. The experiments were independently repeated four times, and the mean of 4 experiments (*n* = 4) is shown. Data are presented as mean ± SEM.

**Additional file 3.** Recombinant mouse CCL1 induces chemotactic migration of macrophages, in vitro. (A) The number of F4/80^+^ cells that crossed to the lower surface of the membrane in control medium (MEM with 1% FBS) with and without addition of rmCCL1. (B) The number of F4/80^+^ cells that crossed to the lower surface of the membrane in the CM of vehicle-treated astrocyte with and without addition of rmCCL1.

**Additional file 4. **Direct effects of ODN 2088 on macrophage polarization, in vitro. (A) ODN 2088 does not counteract the effects of IFN-γ on macrophages. Macrophage cultures were treated with 20 ng/ml IFN-γ for 24 h, in the presence of vehicle or 1 μM ODN 2088. There were no statistical differences in the number of F4/80^+^/iNOS^+^ double-labeled cells [*p* = 0.425, independent-sample *t*-test, two-tailed]. (B) Direct antagonism of macrophage TLR9 does not alter the percentage of F4/80^+^/Arg-1^+^ cells. Macrophage cultures were treated with vehicle or 1 μM ODN 2088 for 24 h. The percentage of the F4/80^+^/Arg-1^+^ cells in the macrophage cultures did not indicate statistical differences between the two groups [*p* = 0.823, independent-sample *t*-test, two-tailed]. (C) Direct antagonism of TLR9 in macrophage cultures exposed to vehicle-treated astrocyte CM did not alter the percentage of F4/80^+^/Arg-1^+^ double-labeled cells. No statistical differences between the two groups were observed [*p* = 0.974, independent-sample *t*-test, two-tailed]. The experiments were independently repeated twice, yielding similar results. Results from a representative experiment are shown. Data are presented as mean ± SEM.

**Additional file 5.** Identification of M1 and M2 macrophages at the lesion core following SCI. (A) A scheme delineating the lesion core and 500-μm wide glial scar region adjacent to the lesion core. (B) A representative fluorescent image showing the F4/80 (green) and iNOS (red) double-labeled cells at the lesion core. Arrows point at examples of F4/80^+^/iNOS^+^ double-labeled cells. (C) A representative fluorescent image showing F4/80 (green) and Arg-1 (red) double-labeled cells at the lesion core. Arrows point at examples of F4/80^+^/Arg-1^+^ double- labeled cells. The sections in A and B are counter stained with DAPI (blue). (D) A representative fluorescent image showing the glial scar double-labeled with F4/80 (green) and iNOS (red), and counter stained with DAPI (blue). Note the absence of iNOS immunoreactivity in F4/80 cells (arrows) at the glial scar. Scale bar: 10 μm.

**Additional file 6. **Effects of ODN 2088 on TGF-β1 release by SC astrocyte, in vitro. Quantification of TGF-β1 levels in CM of ODN 2088- or vehicle-treated astrocytes [**p < 0.01, independent-sample *t*-test, two-tailed]. The experiment was independently repeated four times, yielding similar results. Results from a representative experiment are shown. Data are presented as mean ± SEM.

**Additional file 7. **Effects of IgG_2A_ isotype control on macrophage polarization, in vitro. Macrophage cultures were exposed to (A) vehicle- or (B) ODN 2088-treated astrocyte CM for 24 h, in the presence or absence of 1 μg/ml IgG_2A_ isotype controls. Graphs show the quantification of the F4/80^+^/Arg-1^+^ double-labeled cells [*p* = 0.4376 and *p* = 0.1892 for A and B, respectively, by independent-sample *t*-test, two-tailed]. The experiments were independently repeated twice, yielding similar results. Results from a representative experiment are shown. Data are presented as mean ± SEM.

**Additional file 8.** Graphs showing results obtained with biological repeats in different experiments. Two additional biological repeats corresponding to the experiments shown in Fig. 4e (A), Fig. 5d (B and C), Fig. 7a (D), Fig. 7b (E), Fig. 7c (F), and Fig. 7d (G).

## Data Availability

The datasets used and/or analyzed during the current study are available from the corresponding author on reasonable request.

## Abbreviations

AraC: Cytosine-1-β-D-arabinofuranoside
Arg-1: Arginase-1
BMS: Basso Mouse Scale
CCL: Chemokine (C-C motif) ligand
CCR: C-C chemokine receptor
CM: Conditioned medium
CNS: Central nervous system
CXCL: Chemokine (C-X-C motif) ligand 1
DAMP: Danger-associated molecular patterns
DAPI: 4′,6-Diamidino-2-phenylindole
EV: Extracellular vesicle
GFAP: Glial fibrillary acidic protein
i.p.: Intraperitoneal
Iba-1: Ionized calcium binding adapter molecule-1
IFN: Interferon
IL: Interleukin
iNOS: Inducible nitric oxide synthases
LP: Lumbar puncture
LPS: Lipopolysaccharide
M-CSF: Macrophage colony stimulating factor
NGS: Normal goat serum
OCT: Optimal cutting temperature
ODN: Oligodeoxynucleotide
p.i.: Post-injury
PAMP: Pathogen-associated molecular patterns
PRR: Pattern recognition receptors
RT: Room temperature
SC: Spinal cord
SCI: Spinal cord injury
SEM: Standard error of the mean
TGF: Transforming growth factor
TLR: Toll-like receptor
TNF: Tumor necrosis factor
