# Supplementary figures and images for: Astroglial TLR9 antagonism promotes chemotaxis and alternative activation of macrophages via modulation of astrocyte-derived signals: implications for spinal cord injury
Source: J Neuroinflammation. 2020 Feb 25;17:73. doi: 10.1186/s12974-020-01748-x (PMC7041103; doi:10.1186/s12974-020-01748-x)

**A**

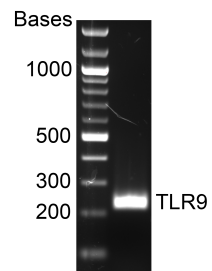

**B**

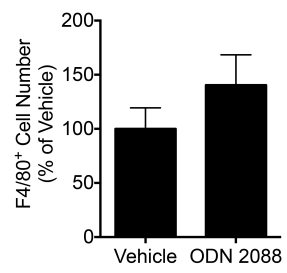

Supplement: Supplementary file 1 — Additional file 1. ODN 2088 does not induce chemotaxis of peritoneal macrophages in the absence of astrocytes. (A) TLR9 expression in macrophages. A gel showing TLR9 transcripts in mouse peritoneal cell cultures by qRT-PCR. (B) The number of F4/80+ cells that crossed to the lower surface of the insert membrane when peritoneal cells were exposed to ODN 2088 in the absence of astrocytes or astrocyte CM [p = 0.2631, independent-sample t-test, two-tailed]. The experiment was independently repeated six times, and the mean of six experiments (n = 6) is shown. Data are presented as mean ± SEM. [file 12974_2020_1748_MOESM1_ESM.pdf]

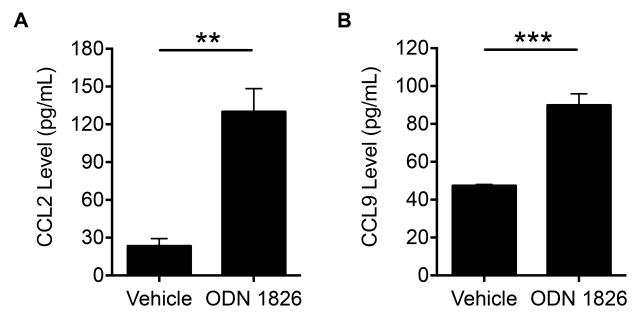

Supplement: Supplementary file 2 — Additional file 2. ODN 1826 increases CCL2 and CCL9 release by astrocytes, in vitro. (A) Quantification of CCL2 levels in CM obtained from vehicle- or ODN 1826-treated astrocytes [**p < 0.01, independent-sample t-test, two-tailed]. (B) Quantification of CCL9 levels in CM obtained from vehicle- or ODN 1826-treated astrocytes [***p < 0.001, independent-sample t-test, two-tailed]. The experiments were independently repeated four times, and the mean of 4 experiments (n = 4) is shown. Data are presented as mean ± SEM. [file 12974_2020_1748_MOESM2_ESM.pdf]

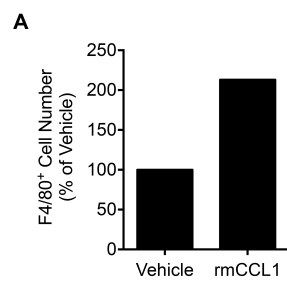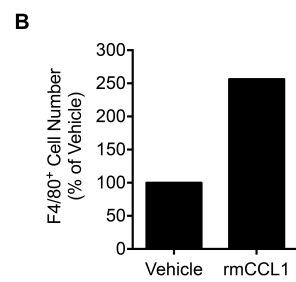

Supplement: Supplementary file 3 — Additional file 3. Recombinant mouse CCL1 induces chemotactic migration of macrophages, in vitro. (A) The number of F4/80+ cells that crossed to the lower surface of the membrane in control medium (MEM with 1% FBS) with and without addition of rmCCL1. (B) The number of F4/80+ cells that crossed to the lower surface of the membrane in the CM of vehicle-treated astrocyte with and without addition of rmCCL1. [file 12974_2020_1748_MOESM3_ESM.pdf]

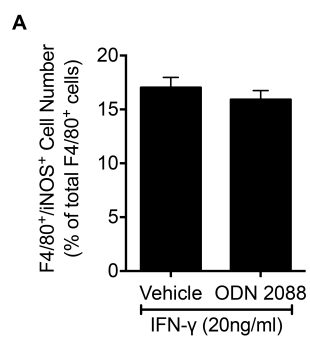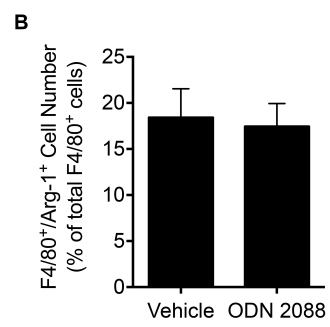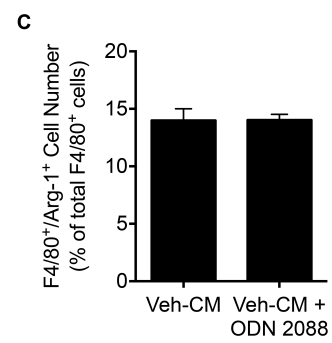

Supplement: Supplementary file 4 — Additional file 4. Direct effects of ODN 2088 on macrophage polarization, in vitro. (A) ODN 2088 does not counteract the effects of IFN-γ on macrophages. Macrophage cultures were treated with 20 ng/ml IFN-γ for 24 h, in the presence of vehicle or 1 μM ODN 2088. There were no statistical differences in the number of F4/80+/iNOS+ double-labeled cells [p = 0.425, independent-sample t-test, two-tailed]. (B) Direct antagonism of macrophage TLR9 does not alter the percentage of F4/80+/Arg-1+ cells. Macrophage cultures were treated with vehicle or 1 μM ODN 2088 for 24 h. The percentage of the F4/80+/Arg-1+ cells in the macrophage cultures did not indicate statistical differences between the two groups [p = 0.823, independent-sample t-test, two-tailed]. (C) Direct antagonism of TLR9 in macrophage cultures exposed to vehicle-treated astrocyte CM did not alter the percentage of F4/80+/Arg-1+ double-labeled cells. No statistical differences between the two groups were observed [p = 0.974, independent-sample t-test, two-tailed]. The experiments were independently repeated twice, yielding similar results. Results from a representative experiment are shown. Data are presented as mean ± SEM. [file 12974_2020_1748_MOESM4_ESM.pdf]

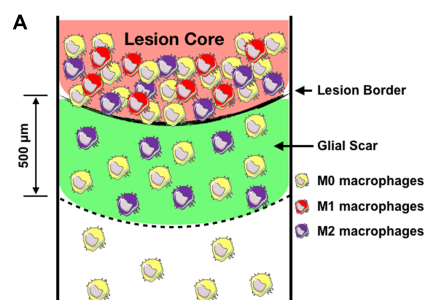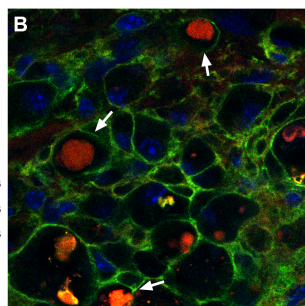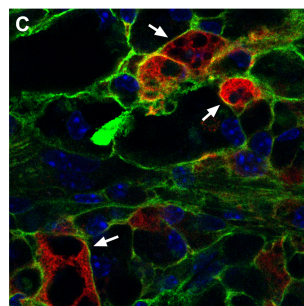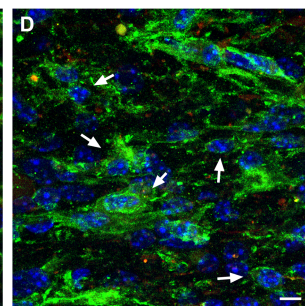

Supplement: Supplementary file 5 — Additional file 5. Identification of M1 and M2 macrophages at the lesion core following SCI. (A) A scheme delineating the lesion core and 500-μm wide glial scar region adjacent to the lesion core. (B) A representative fluorescent image showing the F4/80 (green) and iNOS (red) double-labeled cells at the lesion core. Arrows point at examples of F4/80+/iNOS+ double-labeled cells. (C) A representative fluorescent image showing F4/80 (green) and Arg-1 (red) double-labeled cells at the lesion core. Arrows point at examples of F4/80+/Arg-1+ double- labeled cells. The sections in A and B are counter stained with DAPI (blue). (D) A representative fluorescent image showing the glial scar double-labeled with F4/80 (green) and iNOS (red), and counter stained with DAPI (blue). Note the absence of iNOS immunoreactivity in F4/80 cells (arrows) at the glial scar. Scale bar: 10 μm. [file 12974_2020_1748_MOESM5_ESM.pdf]

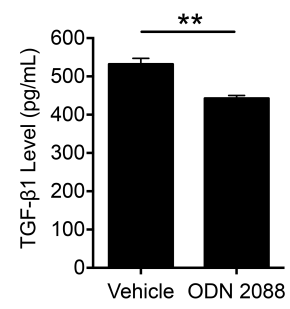

Supplement: Supplementary file 6 — Additional file 6. Effects of ODN 2088 on TGF-β1 release by SC astrocyte, in vitro. Quantification of TGF-β1 levels in CM of ODN 2088- or vehicle-treated astrocytes [**p < 0.01, independent-sample t-test, two-tailed]. The experiment was independently repeated four times, yielding similar results. Results from a representative experiment are shown. Data are presented as mean ± SEM. [file 12974_2020_1748_MOESM6_ESM.pdf]

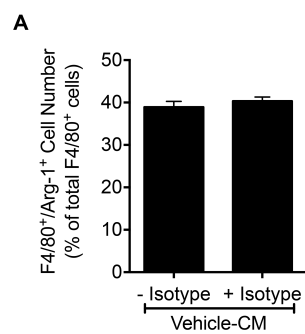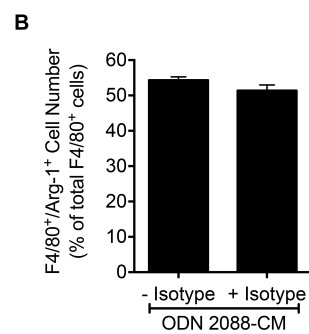

Supplement: Supplementary file 7 — Additional file 7. Effects of IgG2A isotype control on macrophage polarization, in vitro. Macrophage cultures were exposed to (A) vehicle- or (B) ODN 2088-treated astrocyte CM for 24 h, in the presence or absence of 1 μg/ml IgG2A isotype controls. Graphs show the quantification of the F4/80+/Arg-1+ double-labeled cells [p = 0.4376 and p = 0.1892 for A and B, respectively, by independent-sample t-test, two-tailed]. The experiments were independently repeated twice, yielding similar results. Results from a representative experiment are shown. Data are presented as mean ± SEM. [file 12974_2020_1748_MOESM7_ESM.pdf]

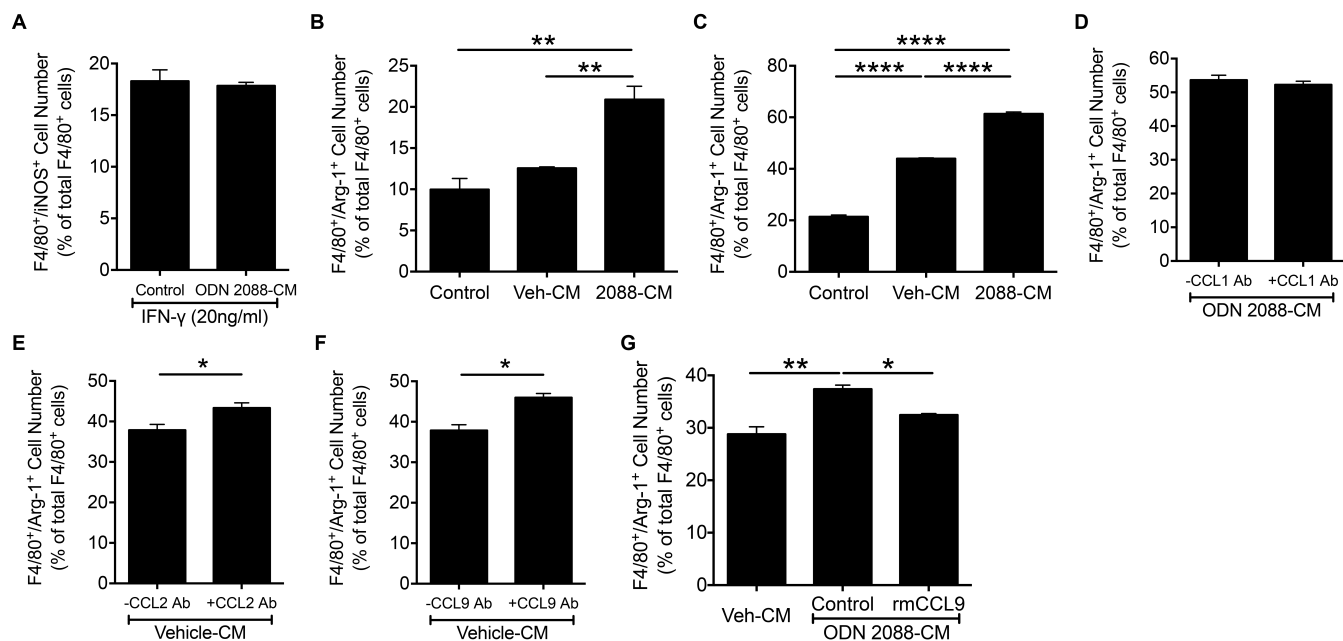

Supplement: Supplementary file 8 — Additional file 8. Graphs showing results obtained with biological repeats in different experiments. Two additional biological repeats corresponding to the experiments shown in Fig. 4e (A), Fig. 5d (B and C), Fig. 7a (D), Fig. 7b (E), Fig. 7c (F), and Fig. 7d (G). [file 12974_2020_1748_MOESM8_ESM.pdf]
